# Energy Dissipation Rate and Micromixing in a Two-Step Micro-Reactor with Intensively Swirled Flows

**DOI:** 10.3390/mi13111859

**Published:** 2022-10-29

**Authors:** Rufat Sh. Abiev, Irina V. Makusheva

**Affiliations:** 1 Ioffe Physical Technical Institute, Russian Academy of Sciences, 194021 St. Petersburg, Russia; 2 Grebenshchikov Institute of Silicate Chemistry, Russian Academy of Sciences, 199034 St. Petersburg, Russia

**Keywords:** microreactor, swirled flows, micromixing, energy dissipation, nanosized particles synthesis

## Abstract

The influence of the hydrodynamics (flow rates *Q*, specific energy dissipation rate ε) on the micromixing in a two-step microreactor with intensively swirled flows (MRISF-2) was studied experimentally. Three methods of liquid input into the reactor were compared: (i) through the upper tangential and axial nozzles (TU1, Ax); (ii) through two upper tangential nozzles (TU1, TU2); (iii) through the upper and lower tangential nozzles (TU1, TL2). Segregation index *X*_s_ used as a measure of micromixing level was determined by means of iodide iodate reaction method. The Bernoulli equation for a device with two inputs and one output was derived to assess the energy consumption. It was revealed that in MRISF-2 up to 99.8–99.9% of input energy is dissipated, i.e., transformed into liquid element deformations thus resulting in better micromixing. For each of three liquid inputs, the dependence ε = *f*(*Q*) could be fairly approximated by an exponent ε = *A*_1_*Q^n^*^1^, with *n*_1_ ≈ 3.0. For connection (TU1, TU2) the dependence *X*_s_ = *f*(ε) falls linearly for *Q* > 2 L/min, but for the low flow rates (*Q* ≈ 1 L/min) there is an unusually small *X*_s_ value; the effect of good micromixing is caused by the kinetic energy concentrated in a small volume of liquid near the neck. The best behavior in terms of micromixing was achieved for the (TU1, Ax) connection scheme: the level of *X*_s_ ≈ 0.01 for ε ≈ 30 W/kg, and comes down with growing ε to *X*_s_ ≈ 0.002 for ε ≈ 30,000 W/kg. These values are 50 and 250 times lower compared to the mixing in a lab glass with a magnetic stirrer, as shown in our previous work. The parameters of dependencies $X_{s}=A_{3}\varepsilon^{n_{3}}$ were found for (TU1, Ax) and (TU1, TL2).

## 1. Introduction

The growing interest in the production of nanosized particles over the past 2–3 decades is due to their special properties and wide areas of application, namely in order to obtain materials with special magnetic properties, catalysts, contrast agents for MRI, and many other applications. The most commonly used synthesis methods are the sol–gel method [1] (and its variants—direct or reverse co-precipitation), the relatively new hydrothermal method [2] and glycine–nitrate combustion [3].

One of the most common direct or reverse co-precipitation methods is fulfilled in a beaker with a magnetic stirrer. The advantage of the “drip” method is its simplicity—no expensive equipment is required; the disadvantage is the uneven macro- and micromixing. It was demonstrated in the work [4] that the use of this method results in a low quality of macro- and micromixing. The low quality of macromixing was approved, for instance, by the 3.5 times excess of the alkali solution (4 M NaOH) to neutralize the 0.05 M HCl solution in the beaker with a magnetic stirrer. The micromixing was found also non-satisfactory (*X*_s_ ≈ 0.5) for a wide range of the angular velocity of the stirrer (from 200 to 1000 rpm).

According to [5] (generalization of data for 8 types of microreactors), micromixing time *t*_m_ is related to the specific energy dissipation rate ε by the dependence

*t*_m_ = 0.15 ε^−0.45^. (1)

Equation (1) demonstrates a significant difference in the micromixing time achieved in microreactors (about 2 ms at ε = 10^4^ W/kg, typical for microreactors) from the micromixing time for reactors with stirrers (420 ms and 150 ms at typical stirrer values ε = 0.1 W/kg and 1.0 W/kg, respectively). These values show that the micromixing time in microreactors is comparable to the reaction time, while for conventional stirred reactors the micromixing time is two orders of magnitude longer. One can conclude that in microreactors, due to the high intensity of micromixing, a state of homogeneity at the molecular or ionic level close to ideal can be achieved [6], which ensures high purity of the formed products, and also (due to short-term exposure) prevents the formation of agglomerates (but subsequent slurry storage and heat treatment should also help to keep the particle size to a minimum).

Thus, both at the macro- and microscale levels, the quality of mixing in laboratory reactors with magnetic stirrers is extremely low, which in many cases explains the difficulties in obtaining high-purity particles in solution synthesis, as well as the reasons for the formation of rather large agglomerates [6].

One of the effective methods for improving the quality of mixing is the use of intensively swirling flows. The combination of intensive swirling with a small volume (no more than 0.5 mL) allows us to achieve high values of ε in the microdevices developed by our group:

- one-step microreactor with intensively swirled flows (MRISF-1) [7];
- two-step microreactor with intensively swirled flows (MRISF-2) [8];
- microreactor with counter swirling flows (MRCSF, both axial and tangential components of velocities have opposite direction) [9];
- microreactor-mixer in opposite swirled flows (MROSF, opposite direction for tangential velocities, adjacent direction of axial velocities) [10].

**Figure 1:**
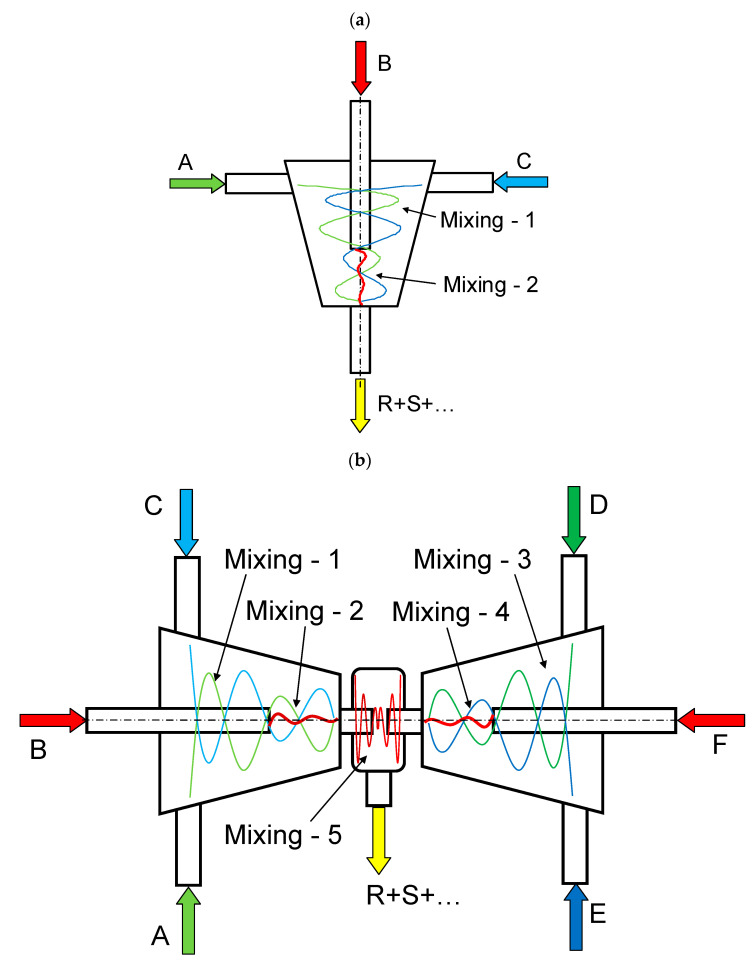
Schematic of the microreactor with intensively swirling flows: MRISF-1 (**a**), MRCSF (**b**).

Examples of microreactors with intensely swirling flows (another name is micro vortex jet apparatuses, micro-VJA or μ-VJA) are shown in Figure 1. A one-step microreactor (MRISF-1, Figure 1a) allows a one-stage process with mixing of two or more solutions (A, B and C); in a two-step microreactor (MRISF-2, Figure 1b), sequential mixing is possible with the formation of an intermediate product (when solutions A, B, and C are mixed), followed by the introduction of high-speed flows of D and E solutions into the apparatus. In a microreactor with counter swirling flows (MRCSF, Figure 1c), three mixing chambers are provided—two conical (each of which is similar to a single-stage reactor), in each of which a fixed temperature and pH value can be kept over time (for example, in one chamber pH = 14, and in the other pH = 5), and a final mixing chamber, in which solutions are mixed with intermediates obtained in first two conical chambers. In each type of microreactor, mixing can occur first in the wide part of the reactor (Mixing-1, Mixing-3 in Figure 1), and then in the narrow part (Mixing-2, Mixing-4 in Figure 1); in the MRCSF, there is also a final mixing chamber in the chamber (Mixing-5 in Figure 1). Our previous studies have shown that the quality of micromixing in a wide part of the reactors is not high enough, and for fast reactions it is expedient to feed reagent solutions into a narrow zone (Mixing-2, Mixing-4 in Figure 1).

Microreactors with swirling flows of reagent solutions developed by our group have the following features:

1. The creation of a powerful swirling flow in a confined space—a small volume of the mixing zone (~0.2–0.5 mL), where the main amount of energy is dissipated, leads to an increase in ε and ensures high homogeneity of the solution;
2. High productivity of the apparatus (up to 10 m^3^ of suspension per day);
3. The possibility of fine individual adjustment of the flow rate of solutions, as well as additional components supplied to the reaction zone (for example, when doping, when obtaining composite materials, etc.);
4. They allow to realize the micromixing time of the order of 1–2 ms.

In [11], the synthesis of calcium fluoride CaF_2_ in a MRCSF was considered. Powders of calcium fluoride doped with ions of rare earth elements are luminophors and are used as precursors for optical ceramics.

In [12], the synthesis of zirconium dioxide in a microreactor with counter swirling flows is discussed. Compared to classical coprecipitation methods, microreactor synthesis exhibits smaller hydrodynamic diameters and reduced agglomeration. According to the data obtained for the synthesis of GdFeO_3_ in a microreactor with impinging jets (MRFIJ) [13], the size of aggregates is 78.5 nm, while the size of aggregates obtained by reverse coprecipitation is 137.4 nm and that of aggregates obtained by direct coprecipitation is 188.2 nm. The calculation showed that the aggregates obtained in MRFIJ contain only 6 crystallites, while those obtained by direct and reverse coprecipitation contain 160 and 31 crystallites, respectively.

**Figure 2:**
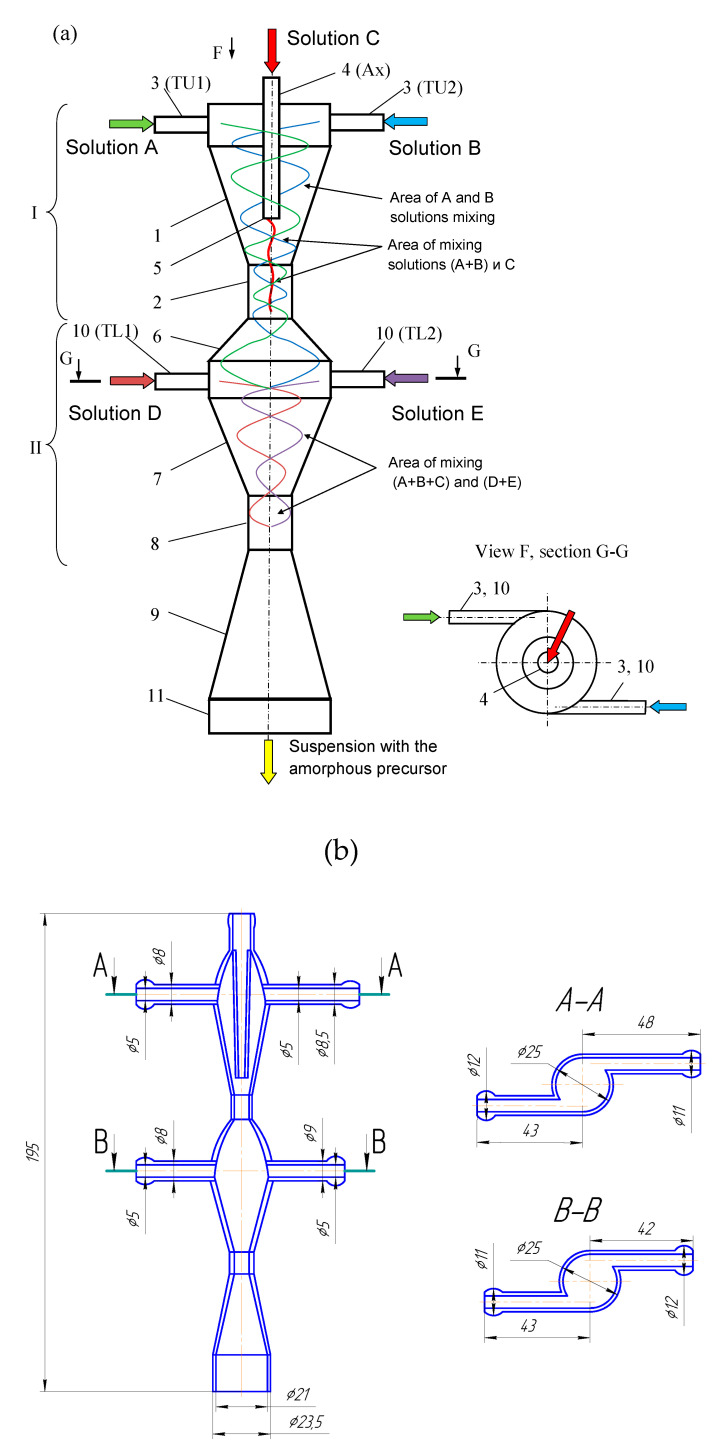
Schematic of two-stage microreactor with intensively swirling flows (**a**) [8] and main sizes of the studied MRISF-2 (in millimeters) (**b**). I—first stage; II—second stage; 1, 7—confusers of I and II steps; 2, 8—necks of I and II steps; 6, 9—diffusers of I and II steps; 3, 10—tangential inlet pipes; 4—axial inlet pipe; 5—nozzle; 11—outlet pipe.

A two-stage (two-step) microreactor with intensively swirling flows (MRISF-2, Figure 2) has the ability to mix two or more solutions at the first stage (solutions A, B, C in Figure 2) while one of the components (C in Figure 2) is introduced through a nozzle directly near the inlet to the neck, i.e., where the rate of dissipation of energy is maximum. At the second stage, several more components can be introduced (solutions D, E in Figure 2) while significantly different pH values can be continuously kept up in the first and second stages, which opens up opportunities for the synthesis of particles with a complex (for example, core-shell) structure, as well as particles of composite materials, the synthesis of which requires a consistent and continuous introduction of components.

Several studies in the field of swirled flows were performed within recent decades [14,15], including those whose results are relevant for chemical engineering [16,17]. Special studies of finite volume and two Lattice Boltzmann solvers with applications to swirled confined flows were conducted in [18]. The study of the gas phase effect on integral characteristics of a swirling two-phase flow with a precessing vortex core is presented in [14], including the formation conditions of a precessing vortex filament. A mathematical model for the swirled non-isothermal flow of two-phase medium with a free surface along the inner wall of a permeable surface has been developed in [15]. Special attention was paid to the use of swirled flows with ferrofluid inside a tube which is equipped with twisted tape in [16]; the effect of magnetic field on the heat transfer and temperature distribution were studied as well. The deposition of particles and droplets in swirled turbulent pipe flow was in the focus of the work [17]; an Eulerian–Lagrangian study based on Direct Numerical Simulation of turbulence was applied. It was shown that swirl may be superimposed to the base flow without disrupting near-wall turbulent structures and their regeneration mechanisms. It was shown in [17] that an optimal synergy between swirl and wall turbulence can be identified to promote separation of particles and droplets.

At present, the two-stage microreactor with intensively swirling flows has been insufficiently studied, which prevents its wide implementation both at the laboratory and industrial level.

The objective of this work is to study the effect of the specific energy dissipation rate on the quality of micromixing in a two-stage microreactor with intensively swirling flows (MRISF-2) and to determine the most efficient operating mode and method for supplying reagent solutions.

To study micromixing, the iodide–iodate technique was used [5]; to determine the specific rate of energy dissipation, the liquid flow rates and pressure losses for each flow were measured and energy losses were calculated according to the Bernoulli equation, which was specially developed for a system with two inputs and one output, (see Appendix A).

Further in the text for the sake of simplicity the solutions could be referred to as “liquids”.

## 2. Materials and Methods

The main dimensions of the studied MRISF-2 were: the inner diameters of input pipes 5 mm, the diameters of output pipes 21 mm, the first neck diameter 6.5 mm, the second neck 8.0 mm. The total height of the device was 195 mm (including connecting nipples).

A two-stage microreactor MRISF-2 consists of a housing in the form of confusers 1, 7 connected in series, necks 2, 8 and diffusers 6, 9, tangential branch pipes 3, 4, 10 for supplying solutions to the apparatus and outlet branch pipe 11 for removing products from the apparatus. The housing consists of two steps arranged coaxially with respect to each other. One axial (central) branch pipe 4 (Ax) and two tangential branch pipes 3 (hereinafter referred to as the upper tangential branch pipes, TU1 and TU2) are attached to the upper stage of the apparatus for input of solutions A, B, C into the apparatus. Two tangential nozzles 10 (hereinafter referred to as the lower tangential nozzles, TL1 and TL2) are attached to the lower stage of the apparatus for G and E solutions input.

The solutions were supplied by two gear pumps TOPSFLO Micro Pump Technology (model MG213XKDC24WI) with a nominal flow rate of up to 3500 mL/min and an operating pressure of up to 10 bar. The material of the inner part of the housing is AISI 316L stainless steel, the gears are PEEK, the seals are PTFE.

To determine the volumetric flow rates of solutions, two Badger Meter (M series M-2000, Check Republic) flowmeters with a bore diameter of 8 mm, flow measurement limits of 100–8000 mL/min with a relative measurement error of ±3 were used. Pressure gauges Elemer (Russia) with a relative error of ±0.2% were employed. The outputs of flowmeters and pressure gauges were connected to an analog digital converter L-Card-14–140 (Russia) linked with a laptop equipped by PowerGraph (Russia) software necessary for collecting, recording and processing measured data.

As a tool for studying the quality of micromixing, the iodide–iodate method [5,19,20,21,22,23,24] was chosen.

Studies to determine the quality of micromixing using the iodite–iodate technique were based on the recommendations given in [5,19]. In this work, sets of concentrations are given (Table 1) corresponding to the optimal optical densities that can be measured by means of UV-VIS spectrophotometer SF-2000 (Russia).

The local velocities were varied along with the flow rates, according to continuity equation, and for the range of flow rate from 1.0 to 3.0 L/min in each feeding branch pipe (having diameters of 5 mm) velocities were in the range *v*_1a_ = *v*_1b_ = 0.87–2.53 m/s, whereas in the neck of the MRISF-2 (with diameter of 3 mm) the velocity was in the range *v*_n_ = 4.83–13.58 m/s.

### 2.1. Determination of the Specific Energy Dissipation Rate

The specific energy dissipation rate was determined as follows. The outlet pipe of the microreactor was submerged under the level of the liquid by the depth of h_p_. The manometer readings were taken for three different ways of solutions supplying to the apparatus (see Figure 2):

1. In one tangential an axial inlet pipes (TU1 and Ax);
2. In two tangential inlet pipes (TU1, TU2);
3. In the upper tangential and lower tangential inlet pipes (TU1, TL2).

The specific energy dissipation rate ε (W/kg) was calculated by use of the following equation:

(2) $$\varepsilon =\frac{N}{\rho V},$$

where ρ is the density of solution, kg/m^3^; *V* is the volume of the micromixing zone, m^3^; *N* is the energy dissipation rate (spent on mixing), W.

The volume of the micromixing zone was considered as a sum of the neck volume (*V*_1_ = 0.15 mL) and the zone between the end of the nozzle and the entrance into the neck (*V*_2_ = 0.20 mL), *V*_0_ = *V*_1_ + *V*_2_ = 0.35 mL.

To determine the value of *N* for each case, the Bernoulli equation for a system with two inputs and one output was derived, which has the following form (see derivation in Appendix A):

(3) $$\begin{array}{l} \rho gQ_{1a}\left(z_{1a}+\frac{p_{1a}}{\rho g}\right)+\rho gQ_{1b}\left(z_{1b}+\frac{p_{1b}}{\rho g}\right)+\rho Q_{1a}\alpha_{1a}\frac{v_{1a}^{2}}{2}+\\ +\rho Q_{1b}\alpha_{1b}\frac{v_{1b}^{2}}{2}=\rho Q_{2}\alpha_{2}\frac{v_{2}^{2}}{2}+\rho gQ_{2}\left(z_{2}+\frac{p_{2}}{\rho g}\right)+\rho gQ_{1a}h_{w1a}+\rho gQ_{1b}h_{w1b} \end{array},$$

where *Q_i_*—liquid flow rate, m^3^/s; *z_i_* is the height from the branch pipe to the immersion height of the apparatus, m; *p_i_*—pressure, Pa; *v_i_* is the velocity in the section, m/s; *g* is the gravity acceleration, m/s^2^; *i* takes the following values: 1*a*—first inlet, 1*b*—second inlet, 2—outlet.

Energy fluxes introduced into the apparatus through each of the inlet branches

(4) $$E_{1a}=\rho gQ_{1a}\left(z_{1a}+\frac{p_{1a}}{\rho g}\right)+\rho Q_{1a}\alpha_{1a}\frac{v_{1a}^{2}}{2}$$

(5) $$E_{1b}=\rho gQ_{1b}\left(z_{1b}+\frac{p_{1b}}{\rho g}\right)+\rho Q_{1b}\alpha_{1b}\frac{v_{1b}^{2}}{2}$$

and removed from the device through the outlet branch:

(6) $$E_{2}=\rho Q\alpha_{2}\frac{v_{2}^{2}}{2}+\rho gQ\left(z_{2}+\frac{p_{2}}{\rho g}\right).$$

The power expended on mixing is defined as the difference between the total mechanical energy at the inlet to the apparatus and at the outlet:

(7) $$N_{mix}=E_{1a}+E_{1b}-E_{2}.$$

**Figure 3:**
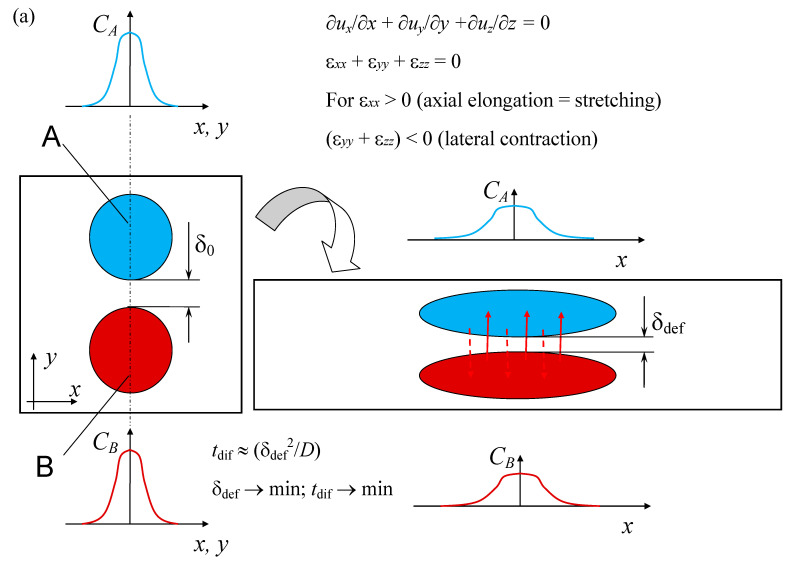

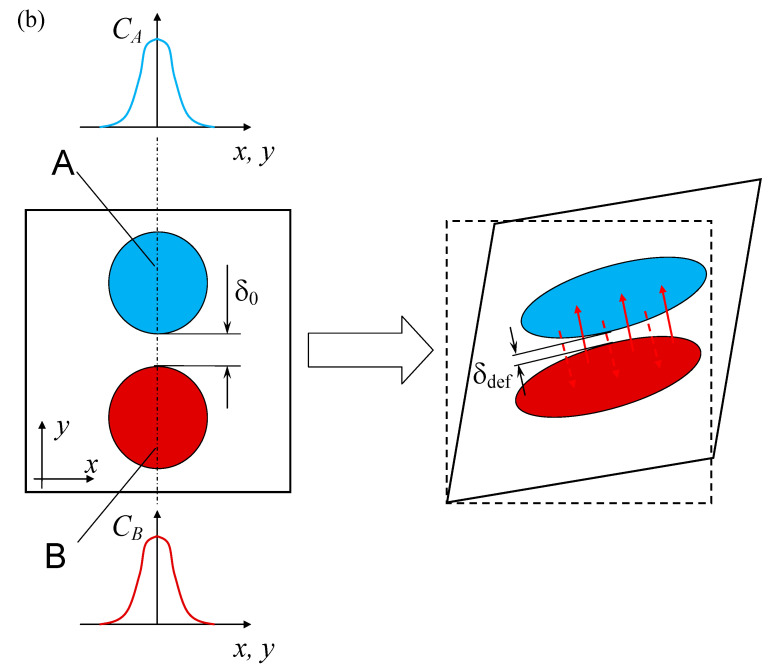
Schematic of the influence of linear (**a**) and angular (**b**) deformations on the process of diffusion of substance spots (concentration spots). The plot is based on the [26] approach.

**Figure 4:**
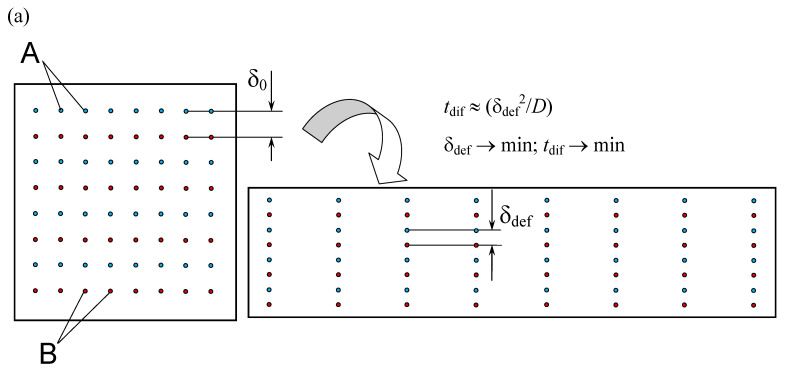

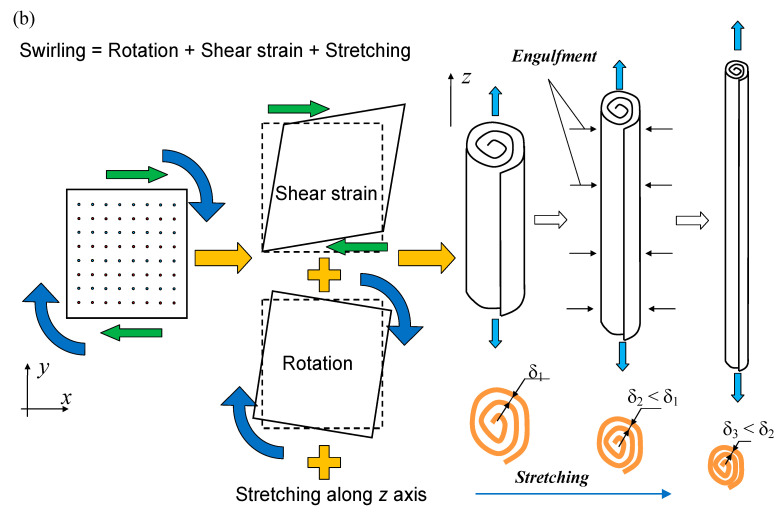
Schematic of the influence of linear (**a**) and angular deformations, as well as swirling (rotation) of the flow (**b**) on the process of diffusion of substances (micromixing), uniformly distributed over the volume.

As is known from fluid mechanics [25], energy dissipation is expressed in linear strain (strain rate tensor components γ_xx_, γ_yy_, γ_zz_) and angular strain (strain rate tensor components γ_xy_, γ_xz_, γ_yz_) of fluid elements. Linear deformations lead to stretching of elements along one axis and narrowing along others, which leads to a decrease in the diffusion path for ions in a liquid. Angular deformations contribute to a spatial shift, resulting in the transfer of ions together with the deforming liquid to new positions and increases the likelihood of contact of ions capable of forming a single molecule (Figure 3 and Figure 4). In addition, in the presence of rotational motion, an intensive movement of ions in space occurs, leading to a sharp increase in the frequency of ion contact; see additional discussion in [26].

To determine the energy efficiency of reactors from the micromixing point of view, the concept of efficiency was proposed, which characterizes the fraction of energy spent on the deformation of the liquid elements *N_mix_* to the total energy introduced into the apparatus *N*_in_ = *E*_1*a*_ + *E*_1*b*_:

(8) $$\eta =\frac{N_{mix}}{E_{1a}+E_{1b}}.$$

### 2.2. Determination of Segregation Index by Use of Iodide–Iodate Method

The iodide–iodate method is based on the use of a system of competing parallel reactions [5,19,20,21,22,23,24]:

quasi-instant neutralization reaction (9)

(9) $$H_{2}{\mathrm{BO}}_{3}^{-}+H^{+}\overset{}{⇄}H_{3}{\mathrm{BO}}_{3}^{}$$

and very fast redox reaction (10):

(10) $${\mathrm{IO}}_{3}^{-}+5I^{-}+6H^{+}\overset{}{⇄}{3I}_{2}+3H_{2}O,$$

as well as accompanying reaction of triiodide formation (iodine can further react with iodide to form triiodide ions I_3_^−^)

(11) $$I_{2}+I^{-}\overset{}{⇄}I_{3}^{-}.$$

The redox reaction (10) is fast, has the same time order as the micromixing process, but much slower than the neutralization reaction (9) [5,19,20].

The iodide–iodate test procedure for determining the quality of micromixing consists of adding a small amount of sulfuric acid to a mixture of solutions of iodide I^−^ and iodate IO_3_^−^ in a buffer solution of H_2_BO_3_^−^/H_3_BO_3_. Under ideal micromixing conditions, the injected acid is instantly dispersed in the reaction medium and immediately consumed by the borate ions H_2_BO_3_^−^ in accordance with the neutralization reaction (9), which is much faster than the redox reaction (10).

If the micromixing time is equal to or greater than the time of the redox reaction, there is a local supersaturation of some microvolumes of the reactor with sulfuric acid, as a result of which the hydrogen ions participating in the reaction (9) with borate ions are able to react with iodide and iodate ions with the formation of iodine I_2_ according to the reaction (10). Thus, the amount of triiodide formed further in the reaction (11) is a measure of fluid segregation.

Borate ions play a dual role: as reagents for the neutralization reaction (9) and as a buffer to maintain a constant pH of the solution. The pH value is selected from a potential pH chart for an aqueous–iodine system. The quality of the results mainly depends on the concentrations of the reagents used, which must be reasonably adapted to the parameters of a particular mixing reactor so that the range of optical density of the resulting solution is in the linearity zone of the spectrophotometer used.

To quantify the quality of micromixing, the segregation index *X*_s_ is used, the value of which is in the range from 0 to 1 [19]. Ideal micromixing is characterized by the value *X*_s_ = 0, for complete segregation *X*_s_ = 1. In the general case, the segregation index is calculated by the following formula [19]:

(12) $$X_{s}=\frac{Y}{Y_{ST}},$$

where *Y* is selectivity for iodine, *Y_ST_*—the iodine selectivity for complete segregation.

Selectivity for iodine denotes the ratio of the number of moles of acid consumed according to reaction (10) to the total number of moles of acid and, provided that the flow rates of the buffer solution and that of sulfuric acid are equal, it is determined in the general case (when sulfuric acid is injected into the reactor) from the equation [27]:

(13) $$Y=\frac{2\left(n_{I_{2}^{\;}}+n_{I_{3}^{-}}\right)}{n_{H_{0}^{+}}}=\frac{2\left(V_{A}+V_{B}\right)\left({[I}_{2}{]+[I}_{3}{}^{-}]\right)}{V_{A}{{[H}^{+}]}_{0}},$$

where *V*_A_ is the volume of the sulfuric acid solution in the reactor, m^3^; *V*_B_ is the volume of the buffer solution in the reactor, m^3^; [I_2_] and [I_3_^−^] are the concentrations of iodine molecules and triiodide ions; [H^+^]_0_ is the initial concentration of hydrogen ions.

With regard to the considered microreactor, the volumes of buffer solution *V*_B_ and sulfuric acid *V*_B_ supplied to the reactor over a period of time Δ*t* are related to their flow rates as follows: *V*_B_ = *Q*_1b_Δ*t*, *V*_A_ = *Q*_1a_Δ*t*, while *Q*_1a_ = *Q*_1b_. This implies *V*_B_ = *V*_A_, and formula (13) takes the form

(14) $$Y=\frac{4\left({[I}_{2}{]+[I}_{3}{}^{-}]\right)}{{{[H}^{+}]}_{0}}.$$

Selectivity for iodine with complete segregation, in turn, is calculated by the formula [21]:

(15) $$Y_{ST}=\frac{{6[\mathrm{IO}}_{3}{}^{-}{]}_{0}}{{{[H}_{2}{\mathrm{BO}}_{3}{}^{-}]}_{0}{+6[\mathrm{IO}}_{3}{}^{-}{]}_{0}},$$

where [IO_3_^−^]_0_ is the initial concentration of potassium iodate; [H_2_BO_3_^−^]_0_ is the initial concentration of boric acid ions.

The numerator of Formula (14) corresponds to the number of moles of acid consumed in the reaction (10) ([I_2_] corresponds to the concentration of iodine that did not pass into triiodide, [I_3_^−^] characterizes that part of I_2_ that was converted to I_3_^−^ in the reaction (11)), the denominator is the total number of moles of acid introduced into the apparatus.

The numerator of Formula (15) is the number of acid moles consumed in the reaction (10), and the denominator is the total number of acid moles consumed in the reactions (9) and (10).

Essentially, the segregation index *X*_s_ reflects the conversion of the acid injected into the reactor, taking into account its participation in the reaction (10), expressed in terms of the concentration of iodine, including iodine partially converted into triiodide I_3_^−^ according to the reaction (11).

The triiodide concentration is determined by the Bouguer–Lambert–Beer law [19,20]:

(16) $${[I}_{3}{}^{-}]=\frac{D}{es},$$

where *D* is the optical density of the sample at a wavelength λ = 353 nm; *s* is the optical length of the beams (in ongoing studies, s = 10 mm); *e* is the molar extinction coefficient of the triiodide ion at λ = 353 nm, *e* = 2395.9 m^2^/mol [19,20].

The concentration of iodine [I_2_] in the solution at the outlet of the reactor is calculated from the quadratic equation:

(17) $$-\frac{5}{3}{\left({[I}_{2}]\right)}^{2}+\left({{[I}^{-}]}_{0}-\frac{8}{3}{[I}_{3}{}^{-}]\right){[I}_{2}]-\frac{{[I}_{3}{}^{-}]}{K_{B}}=0,$$

where *K_B_* is the equilibrium constant depending on the temperature *T* according to the Equation (18) (*T* is in K, *K_B_* is in L/mol).

(18) $$\log_{10}K_{B}=\frac{555}{T}+7.355-2.575\log_{10}T$$

Tests were carried out for two volumes of supplied solutions—200 mL and 500 mL, since when testing with a volume of 200 mL, the results seemed inconclusive. It was revealed that 200 mL is too small an amount to gain the steady state conditions in the microreactor within few seconds of the tests (even for not the highest flow rate of 2.4 L/min the volume of 200 mL was supplied through the MRISF-2 within 200/2400 = 1/12 min = 5 s). Therefore, all the results presented here were obtained at the initial volumes of solutions of 500 mL.

A solution was prepared containing potassium iodide and iodate dissolved in a buffer solution of boric acid and sodium hydroxide, as well as a solution of sulfuric acid with the required concentrations: KIO_3_, [IO_3_^−^] = 0.003 mol/L; [H_3_BO_3_] = 0.045 mol/L; KI, [I^−^] = 0.016 mol/L; [NaOH] = 0.045 mol/L; [H_2_SO_4_] = 0.0198 mol/L; temperature of solutions was 25.5 °C.

Three methods of supplying solutions to the MRISF-2 (Table 2) were studied to determine their effect on energy consumption and the quality of micromixing. In all cases, the volume of the zone of active mixing was taken equal to the sum of the volumes of the neck and the zone in front of it (*V*_0_ = 0.35 mL).

## 3. Results

### 3.1. Results of Determining the Specific Rate of Energy Dissipation

All variables and parameters included in the Bernoulli Equation (3) were calculated. The mean velocities in the nozzles were determined by the formula

(19) $$v_{i}=\frac{Q_{i}}{S_{i}}.$$

Reynolds number was found by equation

(20) Rei=ρvidiμ.

The experimental data obtained for each connection method were approximated by a dependence of the form

(21) $$\varepsilon =A_{1}Q^{n_{1}},$$

where *Q* = *Q*_2_ = *Q*_1*a*_ + *Q*_1*b*_ is the total flow rate through the microreactor.

#### 3.1.1. Liquids Supplied to the MRISF-2 through the Upper Tangential and Axial Branch Pipes (TU1, Ax)

**Figure 5:**
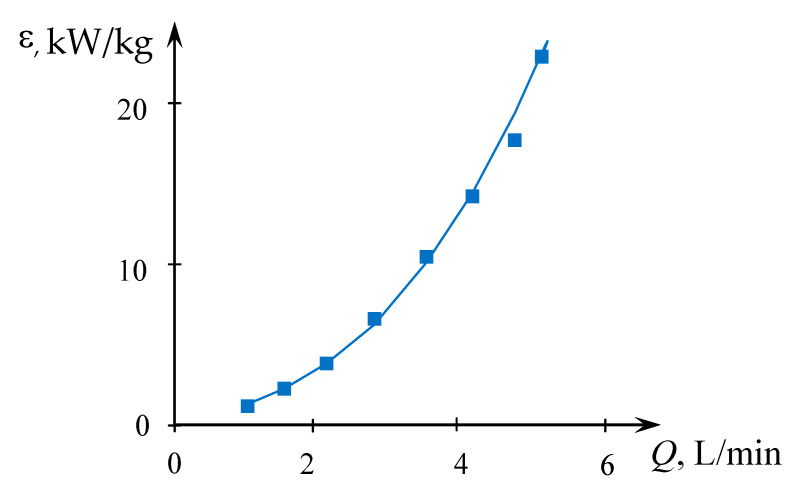
Dependence of the specific energy dissipation rate on the total flow rate (*Q* = *Q*_2_ = *Q*_1*a*_ + *Q*_1*b*_) for solutions supplied through the upper tangential and axial nozzles (TU1, Ax). Points—experimental data, line—dependence $\varepsilon =0.1644Q^{2.973}$(*Q* is in L/min, ε is in kW/kg).

The results of measurements necessary for Equation (3) are presented in the Table S1, the results of calculations are in Table S2 (see Tables S1–S6 in Supplementary materials). The dependence of the specific energy dissipation rate on the total flow rate is shown in Figure 5.

#### 3.1.2. Liquids Supplied to the MRISF-2 through Two Upper Tangential Branch Pipes (TU1, TU2)

**Figure 6:**
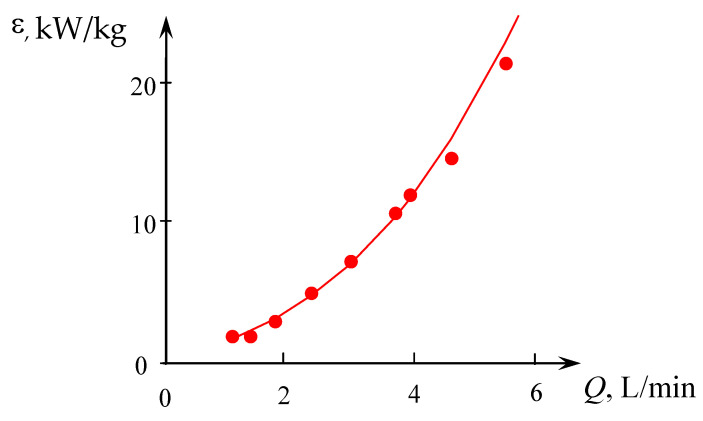
Dependence of the specific energy dissipation rate on the total flow rate (*Q* = *Q*_2_ = *Q*_1*a*_ + *Q*_1*b*_) for solutions supplied through two upper tangential branch pipes (TU1, TU2). Points—experimental data, line—dependence $\varepsilon =0.207Q^{2.732}$ (*Q* is in L/min, ε is in kW/kg).

The results of measurements necessary for Equation (3) are presented in Table S3, the results of calculations are in Table S4. The dependence of the specific energy dissipation rate on the total flow rate is shown in Figure 6.

#### 3.1.3. Liquids Supplied to the MRISF-2 through the Upper Tangential and Lower Tangential Branch Pipes (TU1, TL2)

**Figure 7:**
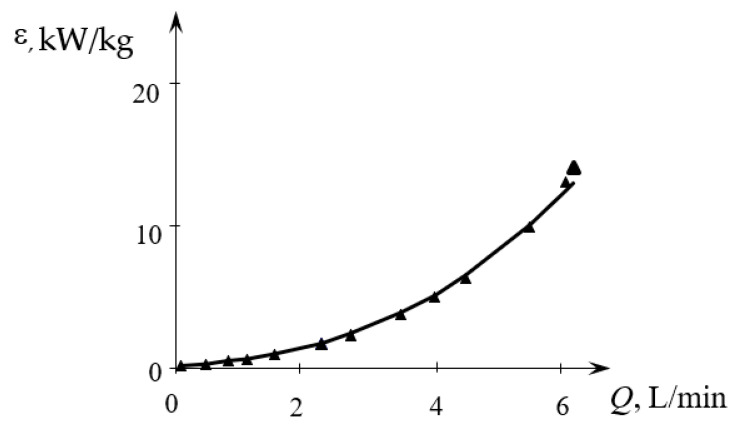
Dependence of the specific energy dissipation rate on the total flow rate (*Q* = *Q*_2_ = *Q*_1*a*_ + *Q*_1*b*_) for liquids supplied through the upper tangential and lower tangential branch pipes (TU1, TL2). Points—experimental data, line—dependence $\varepsilon =0.074Q^{2.846}$ (*Q* is in L/min, ε is in kW/kg).

The results of measurements necessary for Equation (3) are presented in Table S5, the results of calculations are in Table S6. The dependence of the specific energy dissipation rate on the total flow rate is shown in Figure 7.

#### 3.1.4. Comparison of Various Methods of Liquids Input into the MRISF-2

**Figure 8:**
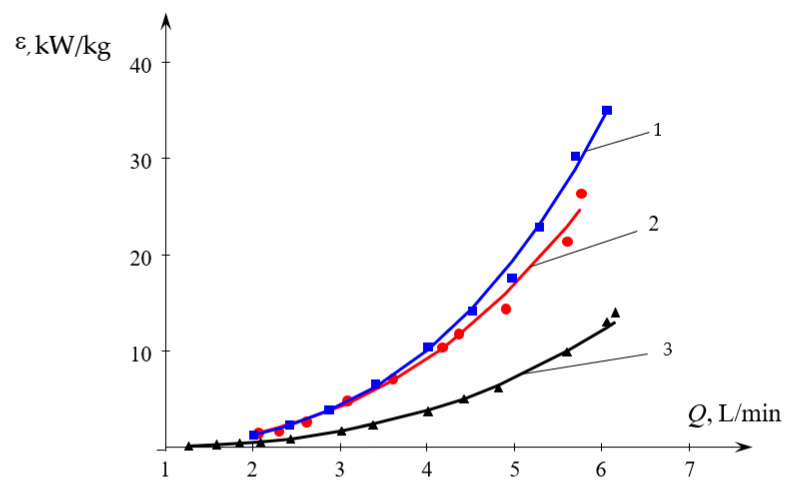
Dependence of the specific energy dissipation rate on the total flow rate (*Q* = *Q*_2_) for various methods of liquids supply into the MRISF-2: 1—(TU1, Ax); 2—(TU1, TU2); 3—(TU1, TL2).

The plot shown in Figure 8 allows to compare the specific energy dissipation rate for various methods of liquids supply into the MRISF-2.

### 3.2. The Results of Determining the Segregation Index

The studies carried out with a solution volume of 200 mL turned out to be unsatisfactory, which manifested itself in the irregular (nonmonotonic) character of the *X*_s_ = *f*(*Q*) curves. Photographing the microreactor during the supply of liquids made it possible to reveal the causes of this phenomenon: (1) at low flow rates, an air-filled zone was formed in the microreactor chamber, the volume of which decreased with time; (2) at high flow rates, during the time of the solutions supply in the microreactor, a stable regime did not have enough time to be established.

For this reason, all experiments on the study of micromixing were rechecked with a volume of solutions of 500 mL; in this case, the dependences *X*_s_ = *f*(*Q*) turned out to be completely monotonic. This paper presents the results obtained only with the solution volumes of 500 mL.

#### 3.2.1. Segregation Index for Liquids Supplied to the Upper Tangential and AXial Nozzles (TU1, AX)

**Figure 9:**
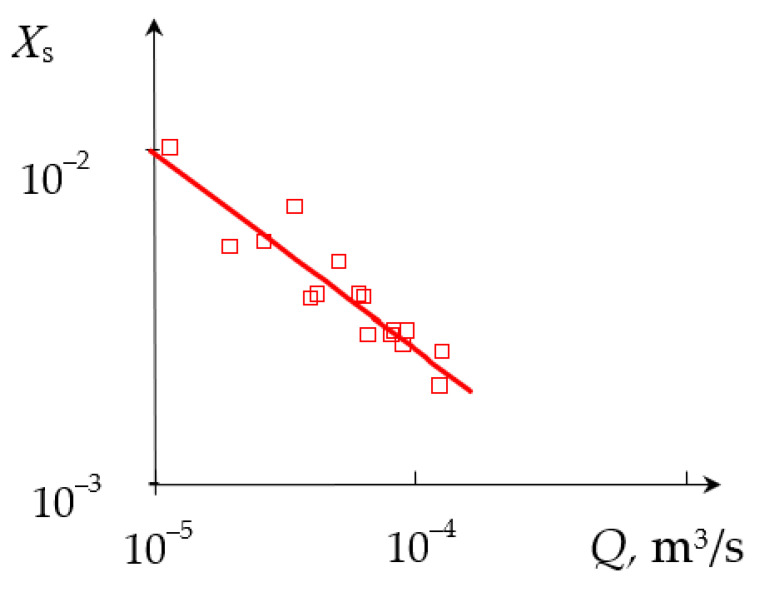
Dependence of the segregation index *X*_s_ on the total flow rate *Q*_2_ (m^3^/s) for liquids supplied to the upper tangential and axial (central) nozzles. Points—experimental data, line—$X_{s}=A_{2}Q^{n_{2}}$, where *A*_2_ = 6.745 × 10^−3^, *n*_2_ = −0.624.

**Figure 10:**
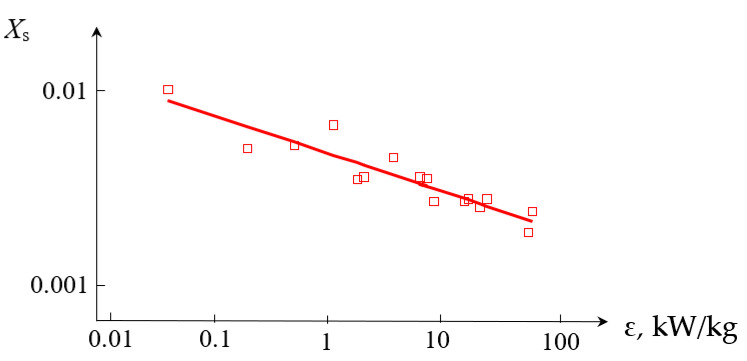
Dependence of the segregation index *X*_s_ on the specific energy dissipation rate ε (kW/kg) for liquids supplied to the upper tangential and axial (central) nozzles. Points—experimental data, line—$X_{s}=A_{3}\varepsilon^{n_{3}}$, where *A*_3_ = 4.618 × 10^−3^, *n*_3_ = −0.21.

The dependencies of the segregation index *X*_s_ on the total flow rate *Q*_2_ and the segregation index *X*_s_ on the specific energy dissipation rate ε for (TU1, AX) connection are shown in Figure 9 and Figure 10, respectively.

#### 3.2.2. Segregation Index for Liquids Supplied to Two Upper Tangential Branch Pipes (TU1, TU2)

**Figure 11:**
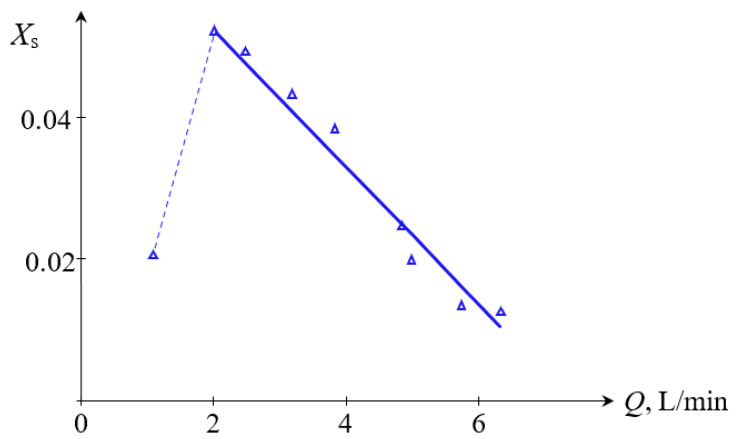
Dependence of the segregation index *X*_s_ on the total flow rate *Q*_2_ (L/min) for liquids supplied to two upper tangential branch pipes (TU1, TU2). Points—experimental data, line—result of approximation for *Q* ≥ 2 L/min: *X*_s_ = *kQ* + *b*, where *k* = – 0.011, *b* = 0.078.

**Figure 12:**
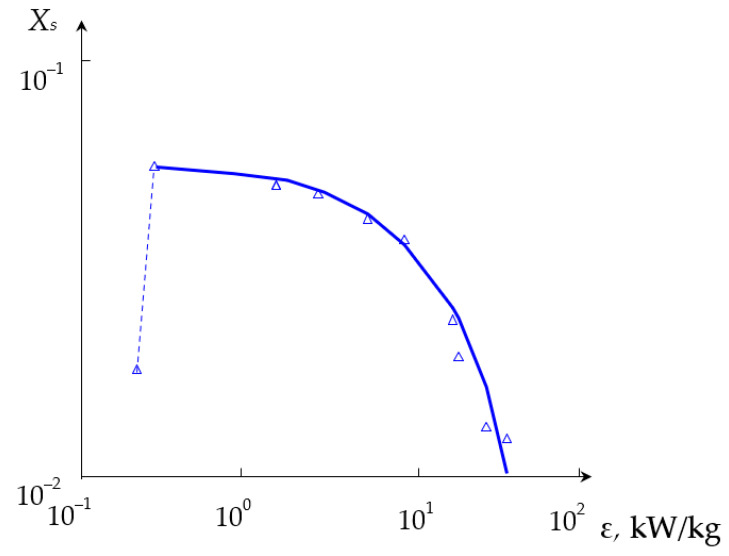
Dependence of the segregation index *X*_s_ on the specific energy dissipation rate ε (kW/kg) for liquids supplied to two upper tangential branch pipes (TU1, TU2). Points—experimental data, line—result of smoothing.

The dependencies of the segregation index *X*_s_ on the total flow rate *Q*_2_ and the segregation index *X*_s_ on the specific energy dissipation rate ε for (TU1, TU2) connection are shown in Figure 11 and Figure 12, respectively.

#### 3.2.3. Segregation Index for Liquids Supplied to the Upper Tangential and Lower Tangential Branch Pipes (TU1, TL2)

**Figure 13:**
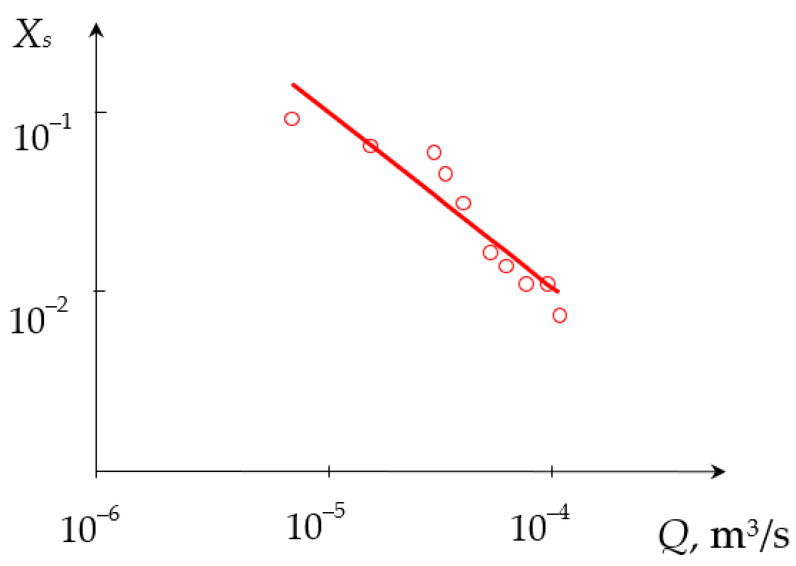
Dependence of the segregation index *X*_s_ on the total flow rate *Q*_2_ (m^3^/s) for liquids supplied to the upper tangential and lower tangential branch pipes (TU1, TL2). Points—experimental data, line—$X_{s}=A_{2}Q^{n_{2}}$, where *A*_2_ = 0.056, *n*_2_ = −0.974.

**Figure 14:**
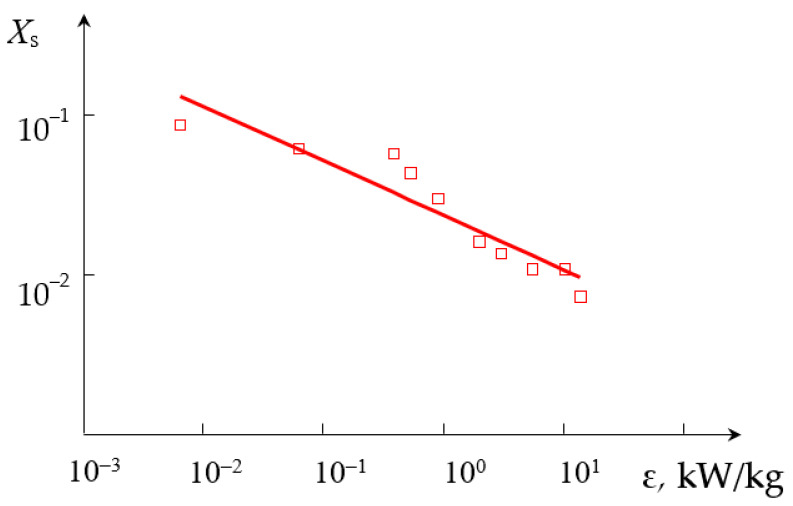
Dependence of the segregation index *X*_s_ on the specific energy dissipation rate ε (kW/kg) for liquids supplied to the upper tangential and lower tangential branch pipes (TU1, TL2). Points—experimental data, line—$X_{s}=A_{3}\varepsilon^{n_{3}}$, where *A*_3_ = 0.023, *n*_3_ = −0.342.

The dependencies of the segregation index *X*_s_ on the total flow rate *Q*_2_ and the segregation index *X*_s_ on the specific energy dissipation rate ε for (TU1, TL2) connection are shown in Figure 13 and Figure 14, respectively.

#### 3.2.4. Generalized Dependencies for Various Liquid Supply Methods

**Figure 15:**
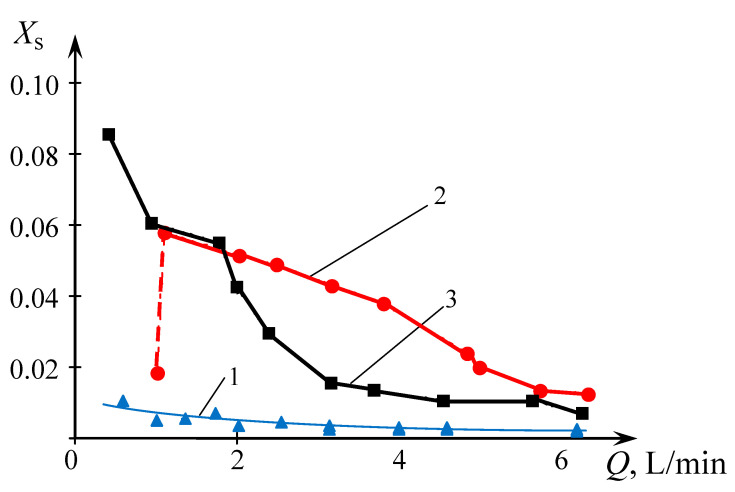
Dependence of the segregation index *X*_s_ on the total flow rate *Q*_2_ (L/min) for various liquids supply methods: 1—(TU1, Ax), 2—(TU1, TU2), 3—(TU1, TL2).

**Figure 16:**
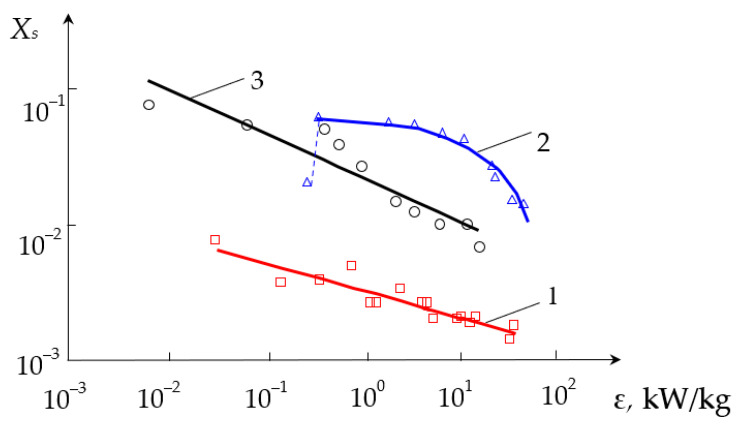
Dependence of the segregation index *X*_s_ on the specific energy dissipation rate ε (kW/kg) for various liquids supply methods: 1—(TU1, Ax), 2—(TU1, TU2), 3—(TU1, TL2).

The dependencies of the segregation index *X*_s_ on the total flow rate *Q*_2_ and the segregation index *X*_s_ on the specific energy dissipation rate ε for all three types of liquid supply are shown in the generalized plots in Figure 15 and Figure 16, respectively.

## 4. Discussion

In all cases, the experimental value for the exponent *n*_1_ in the dependence (21) is close to the theoretical value (*n*_1_ = 3).

The results shown in Figure 8 display that the method of liquid supply into the MRISF-2 plays an important role in the energy dissipation rate. As follows from the results obtained in Section 3.1.4, in all three cases of liquid supply to the MRISF-2, the exponent in Equation (21) is very close to the theoretical value *n*_1_ = 3 (see Table 3). In this case, the line with the maximum value of ε corresponds to the flow through the upper tangential and axial branch pipes (line 1), a little lower is the line corresponding to the flow through the two upper tangential branch pipes (line 2), and line 3 (supply through the upper and lower tangential branch pipes) is substantially lower.

Thus, the specific energy consumption when supplied through pipes (TU1, Ax) (the first method of supply, line 1 in Figure 8) differs little from the supply through pipes (TU1, TU2). This is apparently due to the fact that in both cases the total flow *Q*_2_ passes through both the first and the second necks (pos. 2 and 8 in Figure 2, respectively). Line 1 in Figure 8 lies somewhat higher in relation to line 2, which is due to the additional resistance of the central pipe 4 and nozzle 5 (see Figure 2) when passing through them. In the third way of supply (through pipes TU1, TL2), only flow *Q*_1*a*_ ≈ *Q*_2_/2 passes through the first neck (pos. 2 in Figure 2), and only through the second neck does the full flow *Q*_2_ pass. If we assume that the main hydraulic resistance is concentrated in the zones of each of two necks, and the total drag coefficient of MRISF-2 is equal to ζ, i.e., for the second connection method Δ*p*_2_ = ζ*Q*^2^, then the pressure loss for the third connection method will be the sum of the losses in the first and second necks:

Δ*p*_3_ = ζ/2 (*Q/*2)^2^ + ζ/2 *Q*^2^ = 0.625 ζ*Q*^2^, (22)

and power consumption is

*N*_3_ = ζ/2 (*Q/*2)^3^ + ζ/2 *Q*^3^ = 0.5625 ζ*Q*^3^. (23)

Therefore, according to this estimate, ε_3_/ε_2_ ≈ 0.56. As follows from Figure 8, this ratio is quite satisfactorily confirmed by experimental data. At the same time, the ratio of the coefficients *A*_1_ for the second and third connection methods is somewhat different due to the fact that the exponents *n*_1_ are not exactly equal to 3.

On the other hand, as expected (see e.g., Equation (1)), the higher specific energy dissipation rate results in faster micromixing and better quality of micromixing, as demonstrated in Section 3.2. Some deviations from this general trend (see Figure 11 and Figure 12) are discussed below.

The experimental results for dependence *X*_s_ = *f*(*Q*) were approximated either by exponential trend line (for the connections (TU1, Ax), (TU1, TL2) only)

(24) $$X_{s}=A_{2}Q^{n_{2}},$$

or for the connection (TU1, TU2):

*X*_s_ = *kQ* + *b*. (25)

From the dependence (21) obtained above, it is easy to express the flow rate:

(26) $$Q={\left(\frac{\varepsilon }{A_{1}}\right)}^{\frac{1}{n_{1}}}.$$

Substituting Formula (26) into the expression (24), we obtain the Equation (27) applicable for cases (TU1, Ax) and (TU1, TL2) only:

(27) $$X_{s}=A_{2}{\left(\frac{\varepsilon }{A_{1}}\right)}^{\frac{n_{2}}{n_{1}}}=A_{3}\varepsilon^{n_{3}},$$

where *n*_3_ = *n*_2_/*n*_1_, $A_{3}=A_{2}A_{1}{}^{-n_{3}}$.

In general, the dependence (27) is a good approach to describe the lines $X_{s}=f\left(\varepsilon \right)$ for cases (TU1, Ax) and (TU1, TL2), whereas for the case (TU1, TU2) it has a more complicated character. The values of parameters for Equations (24) and (27) are collected in Table 4, the parameters for Equation (25) are shown in Table 5.

The deviation from the dependence (27) for the case (TU1, TU2) could be attributed to the fact that the flows supplied through two tangential branch pipes TU1 and TU2 simultaneously result in more intensive centrifugal forces. As a result, there are either the empty volumes in both upper and lower chambers filled by air, or some bubbles in the liquid caused by more intensive swirled flow (see photographs in Appendix B).

For *Q*_2_ = 1.0 L/min for the case (TU1, TU2), it was observed that the mixing of two flows starts in the area close to the first neck of the MRISF-2 (see the case *Q*_2_ = 2.0 L/min in Table A1) in Appendix B, resulting in quite good micromixing (*X_s_* ≈ 0.02); similar behavior is observed in the second chamber. In other words, for *Q*_2_ < 2 L/min the volume of the micromixing is small, and therefore the energy dissipation is concentrated in this small area, leading to better micromixing.

For increased flow rates (2 L/min ≤ *Q*_2_ ≤ 4 L/min), the volume of the first chamber is filled with liquid except the most central area around the nozzle where the bubbles are present. The mixing starts already in the wide part of the first chamber where the angular velocity is quite low and the volume of the liquid is large. On the other hand, the contact between liquids (and formation of iodine) occurs in the wide part of the first chamber. This results in both smaller local specific energy dissipation rate and worse segregation index *X*_s_ (up to *X*_s_ ≈ 0.55 for *Q*_2_ = 2 L/min). With increasing flow rates (*Q*_2_ ≥ 4 L/min, see Figure 11), the intensity of mixing grows, leading to higher specific energy dissipation rate and improved micromixing, resulting in decrease of *X*_s_ (down to *X*_s_ ≈ 0.01).

The reason of the worse micromixing for the case (TU1, TL2) at the same flow rates compared to the case (TU1, Ax) is that the main (micro)mixing takes place in the second chamber, where the liquid from the branch pipe TU1 arrives through the first neck, mixing with the second flow, which, in turn, comes from the branch pipe TL2. It is worth noting the mixing occurs in a quite wide volume of the diffuser 6 (see Figure 2) with not the highest rotational velocity, hence the local specific energy dissipation rate ε_loc_ here is lower and segregation index *X*_s_ is almost one order of magnitude higher compared to the case (TU1, Ax) (see Figure 16).

For the case (TU1, Ax), the mixing happens in the area at the tip of nozzle 5 (see Figure 2) and downstream, into highly swirled flow, including the area of the first neck, with the highest local specific energy dissipation rate ε_loc_. In this small area, the injected flow has quite high kinetic energy (both rotational and axial components), necessary to intensively deform liquid elements and to guaranty the best level of micromixing.

The ability to mix triple times for the case (TU1, Ax): (i) at the tip of the nozzle 5; (ii) in the area of the first neck; (iii) in the area of the second neck is evidently a crucial advantage of this liquid supply method for the macromixing.

## 5. Conclusions

The micromixing in a two-step microreactor with intensively swirled flows (MRISF-2) was studied experimentally by use of iodide–iodate reaction method. Three methods of two liquids supply were studied: (i) through the upper tangential and axial nozzles (TU1, Ax); (ii) through two upper tangential nozzles (TU1, TU2); (iii) through the upper and lower tangential nozzles (TU1, TL2).

The specific energy dissipation rate was found to be a function of the flow rate according to the Equation (21), with the exponent close to the theoretical value *n*_1_ ≈ 3.0.

The dependence of the segregation index *X*_s_ on the specific energy dissipation rate ε was revealed to be in good coincidence with the exponential function (27).

The best results were obtained for the connection (TU1, Ax) (*X*_s_ was within 0.01 and 0.002, i.e., very close to the ideal micromixing).

Falk and Commenge [5] on the basis of 8 micromixers studies comparison obtain ε ≈ 10–10^4^ W/kg, and corresponding mixing time was identified as being in the range of *t*_m_ = 0.1 to 0.003 s (see Equation (1)). These values of *t*_m_, on the other hand, are related to segregation index *X*_s_ in the range from 0.01 to 0.0004. This range coincides to a certain extent with results obtained in the presented paper (0.01 to 0.002), especially if we take into account the fact that the sizes of the studied device were in the millimetric range (about 3 mm for the neck of the housing). This result demonstrates that MRISF is comparable with the best micromixers studied in [5].

According to the results obtained in this paper, the advantages of the studied microreactor MRISF-2 could be formulated as follows:

(i). the swirled flow initiated in MRISF-2 allows to obtain micromixing quality comparable with that in other microreactors studied in [5] at the same level of specific energy dissipation rate;
(ii). unlike “smaller” microreactors discussed in [5] (with typical diameters of about 0.1–0.2 mm), the studied MRISF-2 having the diameter of the neck of 3 mm is capable of producing up to 10 m^3^/day of suspension with nanosized particles as it was demonstrated in our previous works [6,11,12]; thus, the transition to the industrial level of production is not an issue for MRISF-2;
(iii). favorable conditions of quite high productivity and excellent quality of micromixing open an attractive perspective to use MRISF-2 in the production of nanosized particles at industrial scale.

The perspectives of this work could be the study of the influence of the tip of the nozzle relative to the neck of the MRISF-2 housing on both the micromixing quality and specific energy dissipation rate. In addition, an extensive CFD study could allow to determine more precisely the areas with the highest level of energy dissipation as well as to optimize the geometry of the device.

## Figures and Tables

**Table 1:** Combinations of reagent concentrations for the experimental determination of the micromixing quality in microreactors recommended in [5,19].

	Reagent Concentration Combination Number					
Concentration, mol/L	1	1b	1c	2	2b	2c
[H^+^]	0.03	0.06	0.04	0.015	0.03	0.02
[KI]	0.032	0.032	0.032	0.016	0.016	0.016
[KIO_3_]	0.006	0.006	0.006	0.003	0.003	0.003
[NaOH]	0.09	0.09	0.09	0.045	0.045	0.045
[H_3_BO_3_]	0.09	0.09	0.09	0.045	0.045	0.045

**Table 2:** Investigated methods of supplying liquids to the MRISF-2.

Three methods of supplying solutions to the MRISF-2		
Into the upper tangential and axial branch pipes (TU1, Ax)	Into two upper tangential branch pipes (TU1, TU2)	Into the upper tangential and lower tangential branch pipes (TU1, TL2)
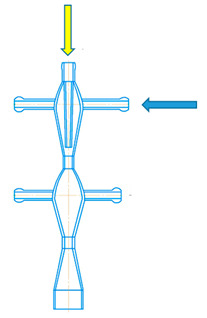	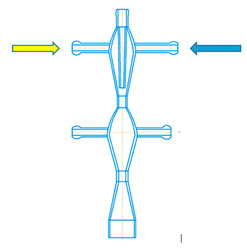	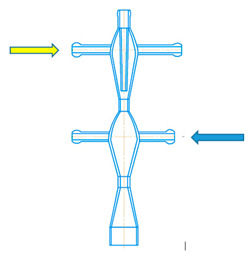

**Table 3:** Parameters of equation (21) for various ways of supplying liquids to MRISF-2 (*Q* is in L/min, ε is in kW/kg).

Parameter Name	(TU1, Ax)	(TU1, TU2)	(TU1, TL2)
*A*_1_	0.1644	0.207	0.074
*n*_1_	2.973	2.732	2.846

**Table 4:** Parameters of Equations (24) and (27) for two ways of supplying liquids to MRISF-2 (*Q* is in L/min, ε is in kW/kg).

Parameter Name	(TU1, Ax)	(TU1, TL2)
*A*_2_	6.745 × 10^−3^	0.056
*n*_2_	–0.624	–0.974
*A*_3_	4.618 × 10^−3^	0.023
*n*_3_	–0.210	–0.342

**Table 5:** Parameters of Equation (25) for various (TU1, TU2) of supplying liquids to MRISF-2 (*Q* is in L/min).

Parameter Name	(TU1, TU2)
*k*	–0.011
*b*	0.078

**Figure A1:**
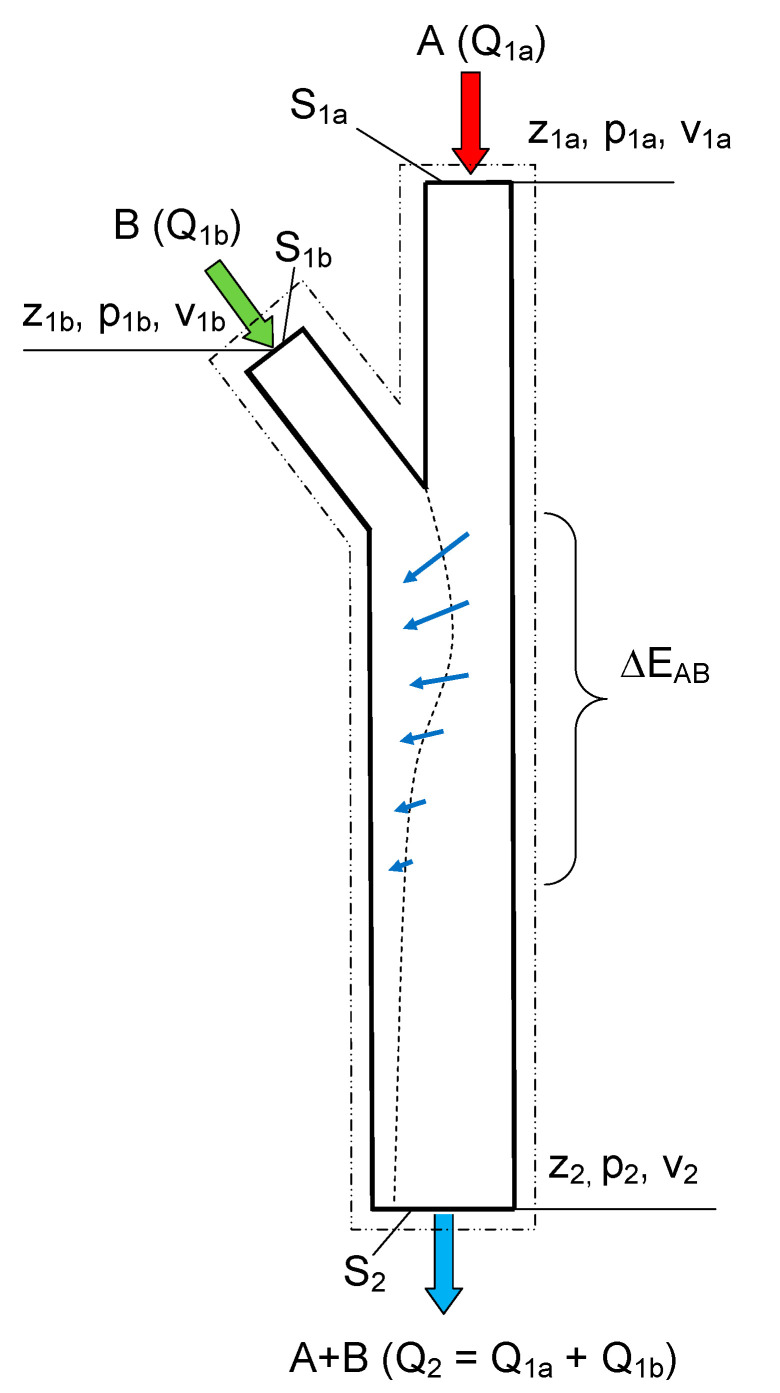
Schematic to the derivation of Bernoulli equation for the system with two inputs and one output. Dashed-dot line is the external energy contour of the device.

## Data Availability

Not applicable.

## Supplementary Materials

The following supporting information can be downloaded at: https://www.mdpi.com/article/10.3390/mi13111859/s1, Figure S1: Microvolumes of the two-stage microreactor MRISF-2 (all data are in mL); Table S1: Results of measurements for solutions supplied to the MRISF-2 through the upper tangential and axial branch pipes (TU1, Ax); Table S2: Results of calculations for solutions supplied to the MRISF-2 through the upper tangential and axial branch pipes (TU1, Ax); Table S3: Results of measurements for solutions supplied to the MRISF-2 through two upper tangential branch pipes (TU1, TU2); Table S4: Results of calculations for solutions supplied to the MRISF-2 through two upper tangential branch pipes (TU1, TU2); Table S5: Results of measurements for solutions supplied to the MRISF-2 through the upper tangential and lower tangential branch pipes (TU1, TL2); Table S6: Results of calculations for solutions supplied to the MRISF-2 through the upper tangential and lower tangential branch pipes (TU1, TL2).

## Appendix A. Derivation of Bernoulli Equation for the System with Two Inputs and One Output

In order to calculate the rate of energy dissipation in the device (apparatus) having two inputs and one output, the following assumptions were taken:

(1) Input flows are steady state, so that no unsteady forces are presented;
(2) Swirling in the output pipe branch is almost exhausted, so that the rotational movement (and corresponding kinetic energy) is negligible compared to the axial flow.

It should also be noted that the energy interchange between two flows introduced through inputs A and B (see the dashed line in Figure A1), e.g., from the flow A to the flow B is the internal energy transfer, and it should not be considered in the total energy balance for the device (because it is just an internal energy exchange and does not cross the external energy contour of the device).

For instance, if the flow A possesses more energy (in relation to B) and transfers some amount of total mechanical (kinetic and potential) energy Δ*E*_AB_ to the flow B (blue arrows in Figure A1), then this energy lost by flow A is received by the flow B, but for the total energy balance related to the external energy contour of the device this amount of energy Δ*E*_AB_ does not play any role due to its internal nature.

It is notable that this energy exchange takes place until two separate flows could be identified, i.e., for the fair mixing it is located in a quite short zone.

For the sake of convenience, both flows A and B could be imagined (formally) as two separate flows (as shown in Figure A1 by the dashed line). Then, Bernoulli equations for each separate flow (i) between sections 1a–2 and (ii) sections 1b–2, respectively, are known from the waste literature as the following:

$$z_{1a}+\frac{p_{1a}}{\rho g}+\alpha_{1a}\frac{v_{1a}^{2}}{2}=z_{2}+\frac{p_{2}}{\rho g}+\alpha_{2}\frac{v_{2}^{2}}{2}+h_{w1a}+\Delta h,$$

$$z_{1b}+\frac{p_{1b}}{\rho g}+\alpha_{1b}\frac{v_{1b}^{2}}{2}=z_{2}+\frac{p_{2}}{\rho g}+\alpha_{2}\frac{v_{2}^{2}}{2}+h_{w1b}-\Delta h,$$

where Δ*h* is the head which corresponds to the internal energy exchange between flows Δ*E*_AB_, as discussed above.

For the arbitrary elementary tubes of flow, one can write:

(i) for *j*th tube of flow between sections 1b–2 in flow A
  (A1) $${\left(p+\rho gz+\frac{\rho u^{2}}{2}\right)}_{j,in}={\left(p+\rho gz+\frac{\rho u^{2}}{2}\right)}_{j,out}+e_{ji}.$$
(ii) for *i*th tube of flow between sections 1a-2 in flow B
  (A2) $${\left(p+\rho gz+\frac{\rho u^{2}}{2}\right)}_{i,in}={\left(p+\rho gz+\frac{\rho u^{2}}{2}\right)}_{i,out}+e_{ij},$$
  where *e_ij_* and *e_ji_* are energy losses in elementary tubes of flow characterizing the internal energy exchange. It should be highlighted that for the total volume of flow *V* (shown by dashed-dot line in Figure A1)
  (A3) $$\int_{V}\left(e_{ij}+e_{ji}\right)\;dV=0,$$
  i.e.,
  (A4) $$\int_{V}e_{ij}\;dV=-\int_{V}e_{ji}\;dV.$$

Energy equation for the liquid without external heat exchange (adiabatic walls):

(A5) $$\frac{dE}{dt}=N=N_{m}+N_{S},$$

where left side (*dE*/*dt*) characterizes energy change and the right side (*N*) is the power of external forces (in Watts), consisting of the power created by mass and surface forces, *N_m_* and *N_S_*, correspondingly:

(A6) $$N_{m}=\int_{V}\vec{u}\cdot \vec{f}\rho dV;$$

(A7) $$N_{S}=\int_{S}\vec{u}\cdot {\vec{p}}_{n}ds=\int_{V}\left[\frac{\partial \left(\vec{u}\cdot {\vec{p}}_{x}\right)}{\partial x}+\frac{\partial \left(\vec{u}\cdot {\vec{p}}_{y}\right)}{\partial y}+\frac{\partial \left(\vec{u}\cdot {\vec{p}}_{z}\right)}{\partial z}\right]dV$$

where $\vec{f}$ is the intensity of the external mass forces ($\vec{f}=\vec{g}$ for the case of gravity); ${\vec{p}}_{n}$ is the full stress tensor (${\vec{p}}_{x}=\sigma_{x}\vec{i}+\tau_{xy}\vec{j}+\tau_{xz}\vec{k}$; ${\vec{p}}_{y}=\tau_{yx}\vec{i}+\sigma_{y}\vec{j}+\tau_{yz}\vec{k}$); ${\vec{p}}_{z}=\tau_{zx}\vec{i}+\tau_{zy}\vec{j}+\sigma_{z}\vec{k}$); *n* is the outward normal to the elementary surface.

For homogeneous (one-phase) liquid without phase changes within the volume of apparatus, the energy of each flow A and B and for the total flow are:

- for the flow A
  (A8) $$E_{A}=\int_{V_{A}}\frac{\rho u^{2}}{2}\;dV+\int_{V_{A}}\rho C_{p}T\;dV+\int_{V_{A}}e_{ji}\;dV,$$
- for the flow B
  (A9) $$E_{B}=\int_{V_{B}}\frac{\rho u^{2}}{2}\;dV+\int_{V_{B}}\rho C_{p}T\;dV+\int_{V_{B}}e_{ij}\;dV,$$
- for the total flow (A + B) the Equations (A8) and (A9) should be summed up:
  (A10) $$\begin{array}{l} E=\int_{V}\frac{\rho u^{2}}{2}\;dV+\int_{V}\rho C_{p}T\;dV+\int_{V}\left(e_{ij}+e_{ji}\right)\;dV=\\ \int_{V}\frac{\rho u^{2}}{2}\;dV+\int_{V}\rho C_{p}T\;dV=E_{k}+E_{D} \end{array}.$$

*E*_k_ and *E*_D_ are the kinetic energy and internal energy, correspondingly. The other types of internal energy are not considered here, as they are almost constant and their time variations are expected to be negligible in the studied circumstances.

By differentiation of Equation (A10), one obtains the left side of Equation (A5), namely:

(A11) $$\frac{dE_{k}}{dt}=\int_{V}\vec{u}\frac{d\vec{u}}{dt}\rho dV=\frac{d}{dt}\int_{V}\frac{\rho u^{2}}{2}\;dV=\int_{V}\frac{\partial }{\partial t}\left(\frac{\rho u^{2}}{2}\right)\;dV+\int_{S}\frac{\rho u^{2}}{2}u_{n}\;dS.$$

From fluid mechanics it is known that the time derivative of the second term in Equation (A10) is linked with dissipative function *D* as follows:

(A12) $$\frac{dE_{D}}{dt}=\frac{d}{dt}\int_{V}\rho C_{p}T\;dV=\int_{V}\mu D\;dV.$$

Dissipative function *D* is known from the classical literature defined as

(A13) $$D=2\left[{\left(\frac{\partial u_{x}}{\partial x}\right)}^{2}+{\left(\frac{\partial u_{y}}{\partial y}\right)}^{2}+{\left(\frac{\partial u_{z}}{\partial z}\right)}^{2}\right]+{\left(\frac{\partial u_{x}}{\partial y}+\frac{\partial u_{y}}{\partial x}\right)}^{2}+{\left(\frac{\partial u_{y}}{\partial z}+\frac{\partial u_{z}}{\partial y}\right)}^{2}+{\left(\frac{\partial u_{z}}{\partial x}+\frac{\partial u_{x}}{\partial z}\right)}^{2}$$

and includes both linear and shear deformations of fluid elements. The meaning of the dissipative function is the part of the mechanical energy which is transformed into the heat leading to the increase of the fluid temperature.

The second term in Equation (A11) is an integral trough total surface *S* which consists of three branch pipes and the side wall: *S* = *S*_n1a_ + *S*_n1b_ + *S*_2_ + *S*_w_.

If one applies the energy Equation (A5) along with Equations (A6), (A7), (A11) and (A12) to the volume shown in Figure A1, one obtains the following results for each term.

For the first term in Equation (A11):

(A14) $$\int_{V}\frac{\partial }{\partial t}\left(\frac{\rho u^{2}}{2}\right)dV=\frac{\rho }{2}\frac{\partial }{\partial t}\int_{l}dl\int_{S}u^{2}dS=\alpha_{0}\frac{\rho }{2}\int_{l}\frac{\partial }{\partial t}\left(v^{2}S\right)dl=\;\alpha_{0}\rho Q\int_{l}\frac{\partial v}{\partial t}dl,$$

where *v* is the superficial velocity, *v* = *v*(*l*), and α_0_ is the coefficient characterizing the non-uniformity of momentum flux:

(A15) $$\alpha_{0}=\frac{\int_{S}u^{2}dS}{v\;Q}.$$

In the studied case, the term expressed by Equation (A14) is considered to be negligible as the flow is steady state.

For the second term in Equation (A11):

- for inlet cross-sections (*S_n_*_1*a*_ and *S_n_*_1*b*_) *un* = −*u*, therefore for *S_n_*_1*a*_
  (A16) $$\int_{S}\frac{\rho u^{2}}{2}u_{n}\;dS=\frac{\rho }{2}\int_{S_{1a}}u^{2}u_{n}dS=-\frac{\rho }{2}\int_{S_{1a}}u^{3}dS=-\frac{\rho }{2}\alpha_{1a}v_{1a}^{3}S_{1a}=-\frac{\rho }{2}\alpha_{1a}v_{1a}^{2}Q_{1a}.$$
  Similarly, for *S_n_*_1*b*_
  (A17) $$\int_{S}\frac{\rho u^{2}}{2}u_{n}\;dS=\frac{\rho }{2}\int_{S_{1b}}u^{2}u_{n}dS=-\frac{\rho }{2}\int_{S_{1b}}u^{3}dS=-\frac{\rho }{2}\alpha_{1b}v_{1b}^{3}S_{1b}=-\frac{\rho }{2}\alpha_{1b}v_{1b}^{2}Q_{1b}.$$
- for outlet cross-section (S2) *u_n_= u*,
  (A18) $$\int_{S}\frac{\rho u^{2}}{2}u_{n}\;dS=\frac{\rho }{2}\int_{S_{2}}u^{2}u_{n}dS=\frac{\rho }{2}\int_{S_{2}}u^{3}dS=\frac{\rho }{2}\alpha_{2}v_{2}^{3}S_{2}=\;\frac{\rho }{2}\alpha_{2}v_{2}^{2}Q_{2}.$$
  Since *u_n_* = 0 on the wall, $\int_{S_{w}}u^{2}u_{n}dS=0$.

Here, the coefficients α_1*a*_, α_1*b*_, α_2_ characterize the non-uniformity of the kinetic energy across corresponding cross-sections (*S_n_*_1*a*_, *S_n_*_1*b*_, *S*_2_):

$$\alpha_{1a}=\frac{\int_{S_{1a}}u^{3}dS}{v_{1a}^{2}Q_{1a}};\;\;\alpha_{1b}=\frac{\int_{S_{1b}}u^{3}dS}{v_{1b}^{2}Q_{1b}};\;\;\alpha_{2}=\frac{\int_{S_{2}}u^{3}dS}{v_{2}^{2}Q_{2}};\;$$

Finally, for the time derivative of kinetic energy of steady state flow one obtains form (A16)–(A18):

(A19) $$\frac{dE_{k}}{dt}=\int_{S}\frac{\rho u^{2}}{2}u_{n}\;dS=\frac{\rho }{2}\left(\alpha_{2}v_{2}^{2}Q_{2}-\alpha_{1a}v_{1a}^{2}Q_{1a}-\alpha_{1b}v_{1b}^{2}Q_{1b}\right).$$

In Bernoulli equation, the term expressed by Equation (A12) is usually presented by the loss head *h*_w_. The total volume of the apparatus could be conditionally divided into two volumes *V*_A_ and *V*_B_, separated by dashed line as shown in Figure A1. It is clear that this line is rather a theoretical border between two flows (because they are in fact well mixed as discussed above, see Equations (A3) and (A4)), but such conception allows to obtain an approach to the separate description of energy losses for two flows. Hence, two energy losses could be defined as follows:

(i) for the flow A
  (A20) $$\frac{dE_{DA}}{dt}=\int_{V_{A}}\rho C_{p}\frac{dT}{dt}\;dV=\rho gQ_{1a}h_{wa},$$
(ii) for the flow B
  (A21) $$\frac{dE_{DB}}{dt}=\int_{V_{B}}\rho C_{p}\frac{dT}{dt}\;dV=\rho gQ_{1b}h_{wb},$$
  and in total:
  (A22) $$\frac{dE_{D}}{dt}=\frac{dE_{DA}}{dt}+\frac{dE_{DB}}{dt}=\rho g\left(Q_{1a}h_{wa}+Q_{1b}h_{wb}\right).$$

Integration of Equation (A6) over volumes *V_A_* and *V_B_* with $\vec{f}=\vec{g}$ yields by

(A23) $$N_{m}=\int_{V_{A}}\rho \vec{u}\cdot \vec{g}dV+\int_{V_{B}}\rho \vec{u}\cdot \vec{g}dV=\int_{S_{1a}}\rho gzudS+\int_{S_{1b}}\rho gzudS-\int_{S_{2}}\rho gzudS.$$

Taking into account that all surfaces in Equation (A7) are flat, i.e., ${\vec{p}}_{n}$ = –*p*, one obtains:

(A24) $$N_{S}=\int_{S}\vec{u}\cdot {\vec{p}}_{n}ds=\int_{S_{1}}pudS-\int_{S_{2}}pudS.$$

By composition, Equations (A23) and (A24) the right side of Equation (A5) could be expressed as:

(A25) $$N=\rho gQ_{1a}\left(z_{1a}+\frac{p_{1a}}{\rho g}\right)+\rho gQ_{1b}\left(z_{1b}+\frac{p_{1b}}{\rho g}\right)-\rho gQ_{2}\left(z_{2}+\frac{p_{2}}{\rho g}\right).$$

Substituting (A19), (A22) and (A25) in Equation (A5) results in the equation

(A26) $$\begin{array}{l} \frac{\rho }{2}\left(\alpha_{2}v_{2}^{2}Q_{2}-\alpha_{1a}v_{1a}^{2}Q_{1a}-\alpha_{1b}v_{1b}^{2}Q_{1b}\right)+\rho g\left(Q_{1A}h_{WA}+Q_{1B}h_{WB}\right)=\\ =\rho gQ_{1a}\left(z_{1a}+\frac{p_{1a}}{\rho g}\right)+\rho gQ_{1b}\left(z_{1b}+\frac{p_{1b}}{\rho g}\right)-\rho gQ_{2}\left(z_{2}+\frac{p_{2}}{\rho g}\right) \end{array}.$$

Finally, after rearrangement of the terms in Equation (A26), the Bernoulli equation for the system with two inputs and one output follows (in Watts):

(A27) $$\begin{array}{l} \underset{I.in.a}{\underset{︸}{\rho gQ_{1a}\left(z_{1a}+\frac{p_{1a}}{\rho g}\right)}}+\underset{I.in.b}{\underset{︸}{\rho gQ_{1b}\left(z_{1b}+\frac{p_{1b}}{\rho g}\right)}}+\underset{II.in}{\underset{︸}{\rho Q_{1a}\alpha_{1a}\frac{v_{1a}^{2}}{2}+\rho Q_{1b}\alpha_{1b}\frac{v_{1b}^{2}}{2}}}=\\ =\underset{I.out}{\underset{︸}{\rho gQ_{2}\left(z_{2}+\frac{p_{2}}{\rho g}\right)}}+\underset{II.out}{\underset{︸}{\rho Q_{2}\alpha_{2}\frac{v_{2}^{2}}{2}}}+\underset{III}{\underset{︸}{\rho gQ_{1a}h_{w1a}+\rho gQ_{1b}h_{w1b}}} \end{array}.$$

The terms *I.in.a* and *I.in.b* in Equation (A27) denote potential energy for inflows A and B, respectively. *I.out*—the same for outflow; the terms designated as *II.in* and *II.out* refer to kinetic energy for inflows and outflow; *III* is the part of mechanical energy irreversibly converted to the deformation of fluid and finally dissipates (see Equations (A12) and (A13)).

In other words, last two terms in (A27) express a full energy dissipation rate (in Watts)

(A28) $$N_{dis}=\rho gQ_{1a}h_{w1a}+\rho gQ_{1b}h_{w1b}.$$

In general, the head loss *h_w_* could be attributed to the total flow rate *Q*_2_ as follows:

(A29) $$N_{dis}=\rho gQ_{2}h_{w}.$$

Hence, *h_w_* is the weighed mean of *h_w_*_1*a*_ and *h_w_*_1*b*_:

(A30) $$h_{w}=\frac{Q_{1a}h_{w1a}+Q_{1b}h_{w1b}}{Q_{2}}.$$

A mean (over the whole volume *V_tot_*) specific energy dissipation rate for the apparatus with two inputs and one output is defined as:

(A31) $$\varepsilon_{mean}=\frac{N_{dis}}{V_{tot}}.$$

Obviously, for each device there is the local volume *V*_0_ within apparatus with the highest level of energy dissipation rate *N_dis_._max_* (for instance, near the throat of the reactor with swirling flows), and in this zone there is the maximal value of specific energy dissipation rate:

(A32) $$\varepsilon_{\max}=\frac{N_{dis.\max}}{V_{0}}.$$

## Appendix B. Photographs of the Flow in MRISF-2 at Various Total Flow Rates for Liquids Supply Method (TU1, TU2)

**Table A1:** Photographs of the flow in MRISF-2 for liquids supply method (TU1, TU2).

Total Flow Rate	Photographs of the Flow
*Q*_2_ = 1.0 L/min	
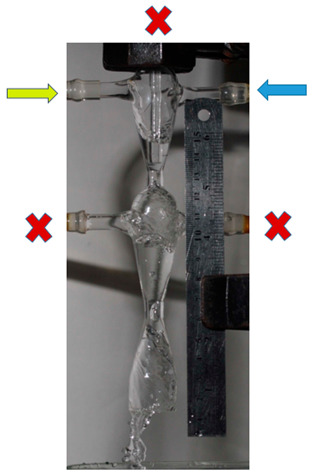
*Q*_2_ = 2.0 L/min	
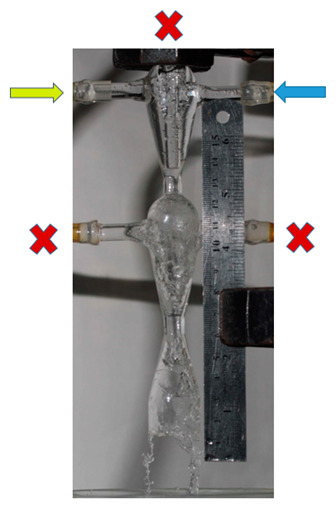
*Q*_2_ = 3.2 L/min	
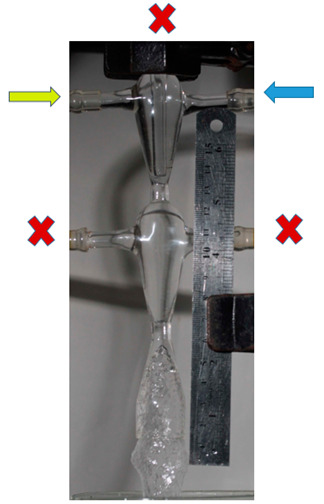
*Q*_2_ = 4.6 L/min	
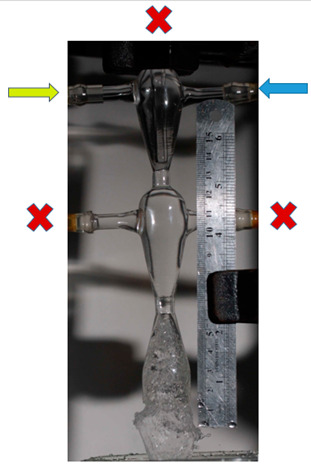
*Q*_2_ = 5.4 L/min	
