# Supplementary material for: Energy Dissipation Rate and Micromixing in a Two-Step Micro-Reactor with Intensively Swirled Flows
Source: Micromachines (Basel). 2022 Oct 29;13(11):1859. doi: 10.3390/mi13111859 (PMC9699309; doi:10.3390/mi13111859)
Supplement: Supplementary file 1 [file micromachines-13-01859-s001.zip › micromachines-1982696-supplementary.pdf]

Supplementary Materials to the Paper

Energy Dissipation Rate and Micromixing in a Two-Step Micro-Reactor with Intensively Swirled Flows

Rufat Sh. Abiev [1,2,*] and Irina V. Makusheva [1,2]

[1] Ioffe Physical Technical Institute, Russian Academy of Sciences, 194021 St. Petersburg, Russia
[2] Grebenshchikov Institute of Silicate Chemistry, Russian Academy of Sciences, 199034 St. Petersburg, Russia
* Correspondence: abiev.rufat@gmail.com

S1. Volumes of the MRISF-2

To measure the (micro)volumes of the microreactors, syringes without a needle with a volume of 20±0.5 mL and 3±0.1 mL were used (hereinafter referred to as a large and a small syringe, respectively).

A large syringe was filled with water to 20 mL, then it was attached to the branch pipe closest to the measured capillary vertically from below (for this, the apparatus was first rotated so that the fitting closest to the measured capillary was located vertically and with the hole down), after that, by pressing the piston, the water was squeezed out until it fills the test volume. The amount of water remaining in the syringe was recorded and then subtracted from the initial value (20 mL).

For a more accurate measurement of some channels and branch pipe, a small syringe was used, which has a more accurate division scale. To do this, the volume preceding these channels was first filled with water from a large syringe, then the outlet from the fitting was plugged for the time that the large syringe was replaced with a small one, already filled with 3 mL, after which the volume of the test capillary was measured.

For some volumes the direct measurements was not possible in this way. To measure them, the following approach was used: first, all the nearest capillaries were measured using the method described above. Then, after the water was completely poured out, the microreactor was again filled with water, so that not

only the zone whose volume was to be measured was filled, but also the nearest capillaries and branch pipes, the volume of which had already been measured in advance. The total volume of the measured area and already measured capillaries and fittings was recorded, after which the values of already known volumes were subtracted.

The total volume of the microreactor was measured similarly. To do this, the side fittings were first plugged, after which the volume was measured in the same way as described above. Results of measurements are shown in Fig. S1.

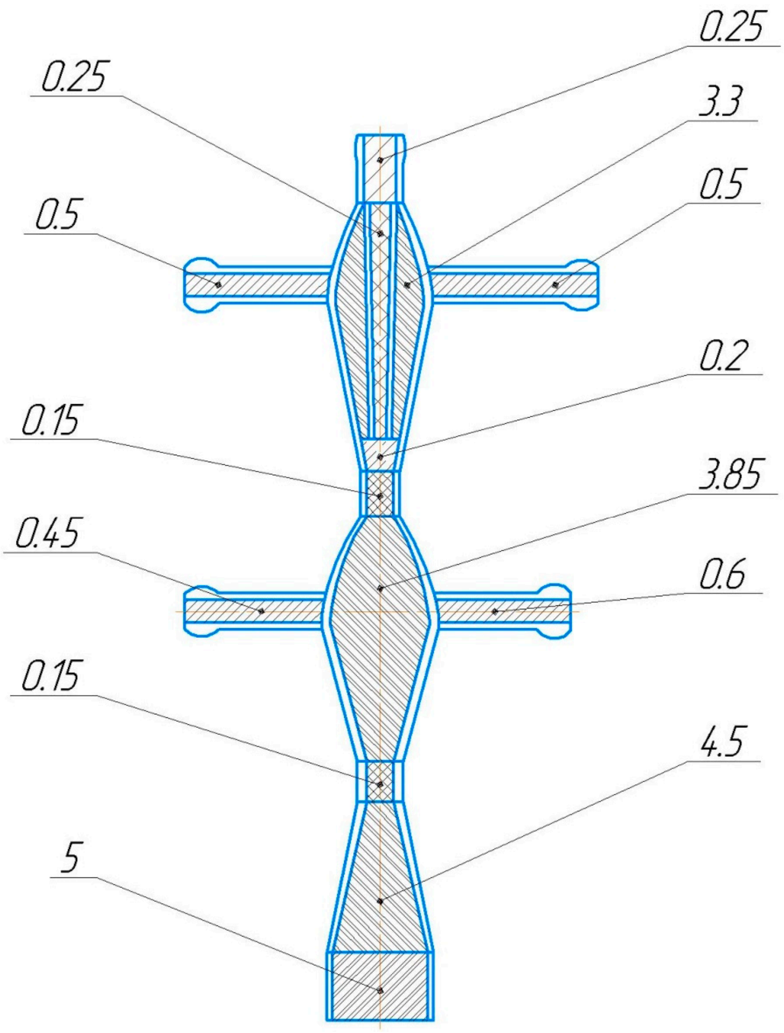

Figure. S1. Microvolumes of the two-stage microreactor MRISF-2 (all data are in mL).

S2. Raw results of measured flow rates and pressure drops in the MRISF-2

Table S1. Results of measurements for solutions supplied to the MRISF-2 through the upper tangential and axial branch pipes (TU1, Ax).

no	Flow rate in the TU1 branch pipe, Q_{1a}, L/min	Flow rate in the Ax branch pipe, Q_{1b}, L/min	Total flow rate, Q_2, L/min	Pressure in the TU1 branch pipe, P_{1a}, kPa	Pressure in the Ax branch pipe, P_{1b}, kPa
1	1.03	0.98	2.01	3.79	17.30
2	1.23	1.18	2.41	6.03	28.22
3	1.45	1.43	2.88	7.57	41.89
4	1.68	1.73	3.41	9.99	61.25
5	1.97	2.03	4.00	13.72	83.45
6	2.28	2.23	4.51	19.73	99.54
7	2.52	2.45	4.97	22.71	111.78
8	2.66	2.62	5.28	25.57	140.00
9	2.86	2.83	5.69	30.77	173.38
10	3.07	2.99	6.06	35.88	186.47

Table S2. Results of calculations for solutions supplied to the MRISF-2 through the upper tangential and axial branch pipes (TU1, Ax).

no	Parameters in inlets		Parameters in outlet		Effici–ency, η, %	ε, kW/kg
	E_{1a}, W (TU1)	E_{1b}, W (Ax)	E_2, W	N_{mix}, W		
1	0.108	0.327	$0.234\cdot 10^{-3}$	0.435	99.94	1.626
2	0.184	0.617	$0.404\cdot 10^{-3}$	0.800	99.95	1.709
3	0.268	1.088	$0.689\cdot 10^{-3}$	1.356	99.95	2.778
4	0.397	1.901	$1.143\cdot 10^{-3}$	2.298	99.95	4.812
5	0.622	3.019	$1.845\cdot 10^{-3}$	3.639	99.95	7.136
6	0.997	3.945	$2.644\cdot 10^{-3}$	4.939	99.95	10.521
7	1.273	4.874	$3.539\cdot 10^{-3}$	6.144	99.94	11.894
8	1.502	6.481	$4.243\cdot 10^{-3}$	7.979	99.95	14.496
9	1.913	8.627	$5.311\cdot 10^{-3}$	10.535	99.95	21.433
10	2.377	9.812	$6.415\cdot 10^{-3}$	12.182	99.95	26.427

Table S3. Results of measurements for solutions supplied to the MRISF-2 through two upper tangential branch pipes (TU1, TU2).

no	Flow rate in the TU1 branch pipe, Q_{1a}, L/min	Flow rate in the TU2 branch pipe, Q_{1a}, L/min	Total flow rate, Q_2, L/min	Pressure in the TU1 branch pipe, P_{1a}, kPa	Pressure in the TU2 branch pipe, P_{1a}, kPa
1	1.024	1.027	2.051	13.32	14.69
2	1.150	1.140	2.290	12.09	13.49
3	1.280	1.320	2.600	18.50	19.80
4	1.569	1.516	3.085	27.90	29.68
5	1.800	1.795	3.595	35.13	38.58
6	2.090	2.070	4.160	45.28	49.11
7	2.200	2.160	4.360	49.05	52.86
8	2.440	2.450	4.890	51.49	57.92
9	2.810	2.781	5.591	68.10	74.23
10	2.781	2.980	5.761	84.70	88.04

Table S4. Results of calculations for solutions supplied to the MRISF-2 through two upper tangential branch pipes (TU1, TU2).

no	Parameters in inlets		Parameters in outlet		Effici–ency, η, %	ε, kW/kg
	E_{1a}, W (TU1)	E_{1b}, W (TU2)	E_2, W	N_{mix}, W		
1	0.271	0.296	$0.249 \cdot 10^{-3}$	0.567	99.96	0.567
2	0.286	0.310	$0.346 \cdot 10^{-3}$	0.596	99.95	0.596
3	0.462	0.507	$0.507 \cdot 10^{-3}$	0.969	99.96	0.969
4	0.833	0.846	$0.846 \cdot 10^{-3}$	1.678	99.95	1.678
5	1.195	1.294	$1.339 \cdot 10^{-3}$	2.488	99.96	2.488
6	1.779	1.891	$2.075 \cdot 10^{-3}$	3.668	99.94	3.668
7	2.027	2.121	$2.389 \cdot 10^{-3}$	4.146	99.94	4.146
8	2.391	2.665	$3.371 \cdot 10^{-3}$	5.053	99.93	5.053
9	3.619	3.859	$5.038 \cdot 10^{-3}$	7.473	99.93	7.473
10	4.344	4.876	$5.512 \cdot 10^{-3}$	9.214	99.94	9.214

Table S5. Results of measurements for solutions supplied to the MRISF-2 through the upper tangential and lower tangential branch pipes (TU1, TL2).

no	Flow rate in the TU1 branch pipe, Q_{1a}, L/min	Flow rate in the TL2 branch pipe, Q_{1b}, L/min	Total flow rate, Q_2, L/min	Pressure in the TU1 branch pipe, P_{1a}, kPa	Pressure in the TL2 branch pipe, P_{1b}, kPa
1	0.625	0.645	1.270	0.829	1.164
2	0.789	0.792	1.581	1.546	2.176
3	0.932	0.925	1.857	2.208	3.515
4	1.054	1.037	2.091	2.805	4.823
5	1.235	1.195	2.430	3.877	6.582
6	1.473	1.540	3.013	5.606	11.510
7	1.480	1.541	3.021	5.696	11.678
8	1.729	1.655	3.384	7.295	13.279
9	2.005	1.999	4.004	9.426	19.112
10	2.230	2.190	4.420	11.640	23.500
11	2.420	2.390	4.810	13.773	26.810
12	2.830	2.770	5.600	18.880	37.800
13	3.065	2.990	6.055	22.570	47.260
14	3.060	3.090	6.150	23.710	50.570

Table S6. Results of calculations for solutions supplied to the MRISF-2 through the upper tangential and lower tangential branch pipes (TU1, TL2).

no	Parameters in inlets		Parameters in outlet		Efficiency, η, %	ε, kW/kg
	E_{1a}, W (TU1)	E_{1b}, W (TL2)	E_2, W	N_{mix}, W		
1	0.028	0.025	$0.059 \cdot 10^{-3}$	0.053	99.89	0.1507
2	0.048	0.047	$0.114 \cdot 10^{-3}$	0.094	99.89	0.2693
3	0.070	0.079	$0.185 \cdot 10^{-3}$	0.148	99.89	0.4252
4	0.094	0.115	$0.264 \cdot 10^{-3}$	0.208	99.87	0.5958
5	0.141	0.174	$0.414 \cdot 10^{-3}$	0.313	99.87	0.8983
6	0.225	0.374	$0.789 \cdot 10^{-3}$	0.597	99.87	1.713
7	0.229	0.379	$0.795 \cdot 10^{-3}$	0.606	99.87	1.737
8	0.336	0.461	$1.117 \cdot 10^{-3}$	0.794	99.86	2.278
9	0.494	0.791	$1.851 \cdot 10^{-3}$	1.281	99.86	3.676
10	0.666	1.055	$2.489 \cdot 10^{-3}$	1.716	99.86	4.923
11	0.843	1.318	$3.208 \cdot 10^{-3}$	2.156	99.85	6.184
12	1.325	2.123	$5.063 \cdot 10^{-3}$	3.439	99.85	9.866
13	1.691	2.823	$6.400 \cdot 10^{-3}$	4.505	99.86	12.920
14	1.745	3.117	$6.706 \cdot 10^{-3}$	4.854	99.86	13.920
